# Expansions and contractions of repetitive DNA elements reveal contrasting evolutionary responses to the polyploid genome shock hypothesis in *Brachypodium* model grasses

**DOI:** 10.3389/fpls.2024.1419255

**Published:** 2024-07-10

**Authors:** María Ángeles Decena, Rubén Sancho, Luis A. Inda, Ernesto Pérez-Collazos, Pilar Catalán

**Affiliations:** ^1^ Escuela Politécnica Superior de Huesca, Universidad de Zaragoza, Huesca, Spain; ^2^ Grupo de Bioquímica, Biofísica y Biología Computacional (Instituto de Biocomputación y Física de Sistemas Complejos (BIFI) Universidad de Zaragoza), Unidad Asociada al Consejo Superior de Investigaciones Científicas (CSIC), Zaragoza, Spain; ^3^ Centro de Investigaciones Tecnológicas y Agroalimentarias de Aragón (CITA), Zaragoza, Spain

**Keywords:** *Brachypodium*, evolution, genome size diversification, polyploid genome shock, repeatome, transposable elements, 5S rDNA loci

## Abstract

*Brachypodium* grass species have been selected as model plants for functional genomics of grass crops, and to elucidate the origins of allopolyploidy and perenniality in monocots, due to their small genome sizes and feasibility of cultivation. However, genome sizes differ greatly between diploid or polyploid *Brachypodium* lineages. We have used genome skimming sequencing data to uncover the composition, abundance, and phylogenetic value of repetitive elements in 44 representatives of the major *Brachypodium* lineages and cytotypes. We also aimed to test the possible mechanisms and consequences of the “polyploid genome shock hypothesis” (PGSH) under three different evolutionary scenarios of variation in repeats and genome sizes of *Brachypodium* allopolyploids. Our data indicated that the proportion of the genome covered by the repeatome in the *Brachypodium* species showed a 3.3-fold difference between the highest content of *B. mexicanum*-4x (67.97%) and the lowest of *B. stacei*-2x (20.77%), and that changes in the sizes of their genomes were a consequence of gains or losses in their repeat elements. LTR-Retand and Tekay retrotransposons were the most frequent repeat elements in the *Brachypodium* genomes, while Ogre retrotransposons were found exclusively in *B. mexicanum*. The repeatome phylogenetic network showed a high topological congruence with plastome and nuclear rDNA and transcriptome trees, differentiating the ancestral outcore lineages from the recently evolved core-perennial lineages. The 5S rDNA graph topologies had a strong match with the ploidy levels and nature of the subgenomes of the *Brachypodium* polyploids. The core-perennial *B. sylvaticum* presents a large repeatome and characteristics of a potential post-polyploid diploidized origin. Our study evidenced that expansions and contractions in the repeatome were responsible for the three contrasting responses to the PGSH. The exacerbated genome expansion of the ancestral allotetraploid *B. mexicanum* was a consequence of chromosome–wide proliferation of TEs and not of WGD, the additive repeatome pattern of young allotetraploid *B. hybridum* of stabilized post-WGD genome evolution, and the genomecontraction of recent core-perennials polyploids (*B. pinnatum*, *B. phoenicoides*) of repeat losses through recombination of these highly hybridizing lineages. Our analyses have contributed to unraveling the evolution of the repeatome and the genome size variation in model *Brachypodium* grasses.

## Introduction

The “polyploid genome shock hypothesis” (PGSH), first proposed by McClintock (1984), postulates rapid genome restructuring following hybridization and whole genome duplication (WGD). PGSH is hypothesized to be a response to the sudden combination of different genomes into a single nucleus, and genetic and epigenetic regulatory adjustments necessary to keep pace between them (Bird et al., 2018; Edger et al., 2019), along with the possible disruption of genome integrity induced by WGD (Scarlett et al., 2023). However, it is less clear how this genomic re-patterning may occur and affect, in the short and long term, the new polyploid genome. Research in some wild and synthetic allopolyploid plants indicates that genomic reshuffling is common in the first generations after WGD, while the allopolyploid genome tends to stabilize and diploidize over time (De Storme and Mason, 2014; Wendel et al., 2018; Mason and Wendel, 2020; Deb et al., 2023). However, other plant allopolyploids have evidenced either long-term genomic instability, perpetuated over generations (Chalhoub et al., 2014; Mason and Wendel, 2020), or a complete absence of genomic restructuring, with the immediate creation of the amphidiploid (Scarlett et al., 2023; Deb et al., 2023; Mu et al., 2023a). One of the components of the genome more severely impacted by the potential restructuring is the repetitive DNA fraction, or repeatome (mobile elements (retrotransposons and transposons), and tandem ribosomal DNA and satellite repeats), which is largely present in the nuclear genome of most plants (Macas et al., 2015; Hidalgo et al., 2017; Pellicer et al., 2018). Three contrasting evolutionary scenarios have been proposed to explain the potential consequences of PGSH on polyploid repeatome turnovers. In some angiosperms, a rapid increase of repeats has been detected in the genomes after rounds of polyploidizations (Chen et al., 2020). In others, the polyploid genomes show repeatome sizes equivalent to those of their diploid progenitor species (McCann et al., 2018). And in other groups, high-level polyploids exhibit a considerable reduction of their repeatome with respect to that of their diploid and low-level polyploid relatives (Chen, 2007; Parisod et al., 2010; Moreno-Aguilar et al., 2022). The ability of centromeric retrotransposon families to proliferate has been interpreted as the potential mechanism for the increased repeatome of the first group of plants, while stabilized post-WGD genome evolution would explain the additive patterns of the second group, and the trend of other repeat families to recombine and lose repeats may have caused the repeatome shrinkage in the third group (Michael, 2014; Chen et al., 2020; Scarlett et al., 2023). Although the proliferation or removal of the repetitive elements from the genomes could have resulted from recombination or double-strand break repair processes (Hawkins et al., 2009; Vu et al., 2017), the driving forces that balance the expansions and contractions of the repeatome are little known (Fedoroff, 2012; Drouin et al., 2021). Analysis of the three alternative evolutionary scenarios of the PGSH has also been hampered by the lack of a suitable specific group to test all their cases.

The importance and impact that the dynamics of repetitive elements has had on genome size (GS) variation and its evolution across the angiosperms has been corroborated in several studies (Dodsworth et al., 2015; Hidalgo et al., 2017; Pellicer et al., 2018). In plants with available reference genomes, the dynamics of transposable elements (TEs) insertions have also been linked to the expression of some core or dispensable genes, which are differentially regulated (Gordon et al., 2017), and to epigenetic effects (Chen, 2007; Fedoroff, 2012; Negi et al., 2016). However, analysis of the repetitive families in most angiosperms that lack assembled and annotated genomes has been performed using genome skimming data and repeatome graphical topology methods (Weiss-Schneeweiss et al., 2015; Garcia et al., 2020; Vitales et al., 2020a; Moreno-Aguilar et al., 2022). The quantification and annotation of repeats in plant genomes relies on the fact that similarity-based clustering of low-coverage genome sequencing reads, which confidentially represents between 0.01 and 0.50 times the coverage of total haploid genome, is proportional to the genomic abundance and length of the corresponding repeat types and could be used to quantify them (Macas et al., 2015; Pellicer et al., 2018; Novák et al., 2020). Furthermore, comparative analysis based on repeat sequence similarities has confirmed the phylogenetic signal of the repeatome in several angiosperm groups (Vitales et al., 2020a, b; Herklotz et al., 2021) and its utility to infer ancestral and recent polyploidization and diploidization events (Moreno-Aguilar et al., 2022). In addition, 5S rDNA graph-based clustering approaches have corroborated the identity of the ancestral progenitor genomes of several polyploid plants (Garcia et al., 2020) and have also uncovered the paleopolyploid nature of current diploidized plant species (Vozárová et al., 2021; Moreno-Aguilar et al., 2022).

The cool seasonal genus *Brachypodium*, consisting of approximately 23 taxa (Catalan et al., 2016; Catalán et al., 2023), has been selected as a model functional system for cereal and biofuel crops and to investigate the evolution of polyploidy in grasses. Annotated reference genomes and considerable genomic resources have been produced for its three annual species (*B. distachyon, B. stacei, B. hybridum*) (Scholthof et al., 2018; Hasterok et al., 2022; Mu et al., 2023a, b; Chen et al., 2024) and for the slender perennial *B. sylvaticum* (Lei et al., 2024). Comparative genomic studies of the annual species evidenced that the allotetraploid *B. hybridum* of recurrent origin did not undergo significant genomic restructuring, showing equivalently inherited parental transposon contents in its two subgenomes (Gordon et al., 2020; Scarlett et al., 2023; Mu et al., 2023a). However, cytogenetic analysis of the less investigated perennial taxa detected considerable differences between the large genome sizes of ancestral polyploids (*B. mexicanum*-4x, 3.7 pg (2C); *B. boissieri*-6x, 3.1 pg) and the relatively small sizes of recently evolved polyploids (e. g., *B. rupestre*-4x, *B. phoenicoides*-4x, 1.4 pg) (Sancho et al., 2022). *Brachypodium* shows a remarkable descending dysploidy trend from ancestral x=10 karyotypes to intermediately-to-recently evolved x=9, x=8 and x=5 karyotypes (Lusinska et al., 2019; Sancho et al., 2022). Phylogenetic subgenome detection algorithms applied to transcriptome data and karyotype barcoding analysis further identified seven diploid subgenomes in the studied *Brachypodium* polyploids, three of them present in extant diploid progenitor species and four orphan (only detected in polyploid species) (Sancho et al., 2022). Except for the thoroughly investigated annual species *B. distachyon*, where transposon landscape analysis revealed high transposable activity of LTR-*Copia* Angela elements and a large contribution to genome size of highly methylated LTR-*Gypsy* Retand elements (Stritt et al., 2020), and the identification of centromeric CRBd retrotransposons elements in six *Brachypodium* species (Li et al., 2018) and the characterization of centromeric species-specific satellite DNA families in the three annual species (Chen et al., 2024), no other study has comprehensively explored the composition and dynamics of repetitive elements across a large representation of *Brachypodium* taxa.

We were particularly interested in using *Brachypodium* as a test case study for the three alternative evolutionary scenarios of PGSH. These *Brachypodium* samples constitute exemplary case studies to investigate the putative role of repeat elements’ dynamics in the evolution of these genomes and to test the potential mechanisms and consequences of the “polyploid genome shock hypothesis” in three different evolutionary scenarios of proliferation, maintenance, and reduction of repeats and genome sizes of allopolyploids that occur within this genus. We also attempted to comparatively analyze the repeatome variations in diploid *Brachypodium* species that show substantial differences in genome sizes (Sancho et al., 2022). The repeatome analysis was also used to assess the potential phylogenetic value of repeat elements in the monotypic Brachypodieae tribe. The objectives of our study were: (i) to characterize and quantify the repetitive elements of 44 representative samples of the main *Brachypodium* species and cytotypic lineages identifying both shared and private repeats; (ii) to analyze the expected correlation between genome size and repeat abundance; (iii) to identify repeat types that could have contributed to the expansions or contractions of the genomes; (iv) to evaluate the phylogenetic signal of repeats using phylogenetic reconstructions; and (v) to assess the three alternative responses to the “polyploid genome shock hypothesis” of *Brachypodium* polyploids and the putative paleo-polyploid origin of some large genome diploids using mobile and tandem repeat data analysis.

## Methods

### Sampling, ploidy levels and genome skimming sequencing

Genomic sequences from 44 accessions of 11 *Brachypodium* species, representing its main out-core (*B. stacei*, *B. mexicanum*, *B. boissieri*, *B. retusum pro partim* (ancestral subgenome), *B. distachyon*, *B. hybridum*), and core-perennial (*B. arbuscula*, *B. sylvaticum*, *B. retusum pro partim* (recent subgenome), *B. pinnatum*, *B. rupestre*, *B. phoenicoides*) lineages (Sancho et al., 2022) were incorporated to the study (**Table 1**; **Supplementary Table S1**; **Supplementary Figure S1**). 41 perennial samples were sequenced *de novo* using genome skimming, while genome data from three annual samples was downloaded from NCBI (*B. distachyon* Bd21-3 (SRR4236817); *B. stacei* ABR114 (SRR3944701); *B. hybridum* ABR113 (SRR3945056; SRR3945058)). We obtained a large cytotype representation of *Brachypodium* through the study of 15 different cytotypes found within these taxa, including diploids, tetraploids and hexaploids (**Table 1**; **Supplementary Table S1**). The samples were classified into species and group-lineages based on previous taxonomic and phylogenetic studies (Schippmann, 1991; Catalan et al., 2016; Díaz-Pérez et al., 2018; Sancho et al., 2022). The 44 selected samples represent all currently recognized evolutionary lineages within *Brachypodium*. They include all the main annual and perennial diploid and polyploid lineages of *Brachypodium*, formed at different evolutionary times and spanning the last 12 Ma (Catalan et al., 2016; Díaz-Pérez et al., 2018; Sancho et al., 2022).

**Table 1:** Samples included in the repeatome analysis of *Brachypodium*. Species, sample’s code, chromosome number (2n), genome size (2C, pg), inferred ploidy level (nx), monoploid genome size (1Cx, pg) and locality of origin.

Species	Code	Chromosome number (2n)	Genome Size (2C/pg)	Ploidy (*n*x)	Monoploid genome (1Cx/pg)	Locality
*B.distachyon*	Bdis_Bd21-3	10	0.631 ± 0	2	0.316	Iraq: Salakudin
*B.stacei*	Bsta_ABR114	20	0.564 ± 0	2	0.282	Spain: Balearic isles
*B. hybridum*	BhybABR113	30	1.265 ± 0	4	0.633	Portugal: Lisbon
*B.arbuscula*	Barb502	18	0.713 ± 0.004	2	0.357	Spain: Canary Isles
*B.boissieri*	Bboi3	48	3.236 ± 0.072	6	0.539	Spain: Granada
	Bboi10	48	3.152 ± 0.04	6	0.525	Spain: Granada
	Bboi15	48	3.149 ± 0.032	6	0.525	Spain: Granada
*B.mexicanum*	Bmex347	40	3.774	4	0.944	Mexico: Hidalgo
	Bmex348H	40*	~3.774*	4*	~0.944*	Mexico: Puebla
	Bmex504	40*	~3.774*	4*	~0.944*	Ecuador: Loja
*B.phoenicoides*	Bpho6-1R	28	1.443 ± 0.019	4	0.361	Spain: Huesca
	Bpho422	28	1.469 ± 0.012	4	0.367	Slovakia: Ružomberok
	Bpho452	38	2.176 ± 0.017	6	0.363	Morocco: Rif Mountains
	Bpho552	38	2.204 ± 0.039	6	0.367	Spain: Cadiz
	Bpho553	38	2.183 ± 0.013	6	0.364	Spain: Malaga
	Bpho554-1	38	2.155 ± 0.02	6	0.359	Spain: Granada
*B.pinnatum*	Bpin505	18	0.822 ± 0.009	2	0.411	Norway: Oslo
	Bpin34	28	1.449 ± 0.018	4	0.362	Great Britain: North Wiltshire
	Bpin514	28	1.537 ± 0.012	4	0.384	Turkey: Samsun
	Bpin520	28	1.499 ± 0.014	4	0.375	Netherlands: Scherpenzeel
*B.retusum*	Bret400	32	1.704 ± 0.024	4	0.426	Spain: Huesca
	Bret407	32	1.715 ± 0.017	4	0.429	Spain: Huesca
	Bret453-4	32*	1.840 ± 0.097	4	0.460	Morocco: Rif Mountains
	Bret454	32*	1.862 ± 0.196	4	0.466	Morocco: Tazza-Bou Idir
	Bret504	32	1.669 ± 0.026	4	0.417	France: Hérault
	Bret555	32	1.715 ± 0.017	4	0.429	Spain: Granada
	Bret403	42	2.373 ± 0.958	6	0.396	Spain: Huesca
	Bret408	42	2.431 ± 0.033	6	0.405	Spain: Navarra
	Bret551	42*	2.109 ± 0.025	6	0.352	Spain: Málaga
	Bret557	42	2.464 ± 0.026	6	0.411	Spain: Cadiz
	Bret561	42	2.362 ± 0.046	6	0.394	Spain: Zaragoza
*B.rupestre*	Brup7	28	1.562 ± 0.016	4	0.391	Russia: Moscow
	Brup439-1	28	1.55 ± 0.022	4	0.388	Spain: Huesca
	Brup441	28	1.483 ± 0.008	4	0.371	Spain: Leon
	Brup442	28	1.56 ± 0.03	4	0.39	Spain: Huesca
	Brup443	28	1.498 ± 0.012	4	0.375	Spain: Guipuzcoa
	Brup444	28	1.492 ± 0.021	4	0.373	Spain: Lugo
	Brup182	38	2.258 ± 0.026	6	0.376	Croatia: Istria
	Brup600	38	2.216 ± 0.013	6	0.369	France: Nans les Pins
	Brup605	38	2.265 ± 0.013	6	0.378	France: Pourrieres
*B.sylvaticum*	Bsyl54-1	18*	0.888 ± 0.008	2	0.444	Morocco: Rif Mountains
	Bsyl466-6	18*	0.928 ± 0.013	2	0.464	Spain: Huesca
	Bsyl477-1	18*	0.932 ± 0.017	2	0.466	Spain: Lerida
	Bsyl501-6	18*	0.947 ± 0.01	2	0.474	France: Alpes Maritimes

Ploidy levels inferred from chromosome counting and genome sizes (this study and previous records). Asterisks indicate the inferred 2n and 2C values and ploidy level of two B. mexicanum samples in accordance with those of their reference taxonomic cytotype based on similar repeat contents. See **Supplementary Table S1** for additional information on taxon authorship, detailed localities and vouchers, and cytogenetic and genomic data sources.

The cytogenetic and karyotypic knowledge of *Brachypodium* has been recently expanded in the recent study of Sancho et al. (2022). Chromosome counting (2n values) analysis was performed on DAPI-stained meristematic root cells following Jenkins and Hasterok (2007). Genome sizes (GS, 2C values) were estimated from fresh leaf tissue using propidium iodide staining of cell nuclei and flow cytometry measurements (Sysmex Ploidy Analyser) following Doležel et al. (2007). Ploidy levels of the *Brachypodium* samples under study were inferred from chromosome and GS estimates obtained in this study and from the repeat data for two samples of *B. mexicanum* (Bmex348H, Bmex504) showing similar repeatome content than their conspecific reference genome sample (Bmex347) (see **Table 1**; **Supplementary Table S1**). Total DNA for 41 (perennial *Brachypodium* samples) out of the 44 samples studied was extracted from fresh and silica gel-dried leaf tissues of plants growing in the common garden of the University of Zaragoza – High Polytechnic School of Huesca and from herbarium specimens (*B. mexicanum* 348H, Herbarium B) (**Supplementary Table S1**). DNA isolation was accomplished using a modified CTAB protocol (Doyle and Doyle, 1987) using ~20mg of tissue. DNA concentration (100-200ng/ul) and absorbance (260/230 nm of 1.8 to 2.1 and 260/280 nm of 1.8 to 2.0) were estimated using Qubit ® 3.0 (Life Technologies, Grand Island, NY) and Biodrop μLITE (Harvard Biosciences), respectively. Genome skimming sequencing was performed from a PCR-free multiplexed pool of KAPA libraries through the Illumina technology in paired-end mode (2 x 101 bp) at the Spanish Centro Nacional de Análisis Genómicos (CNAG, Barcelona). Illumina paired-end reads were checked using FastQC_v0.11.9 (https://www.bioinformatics.babraham.ac.uk/projects/fastqc/). The 41 *Brachypodium* genomic samples used in downstream analysis contained between 10.09 – 30.22 million reads (average 16.7 million reads) with insert sizes ranging between 123 – 329 bp (**Supplementary Table S2**). In addition, sequence data from the three annual *Brachypodium* samples were retrieved from NCBI. The downloaded sequences were filtered using Trimmomatic-0.39 (Bolger et al., 2014) with the following parameters: SLIDINGWINDOW:15:28 (window of bases: quality threshold) and CROP, HEADCROP and MINLEN according to the per-base sequence content and the length of 101 bp, based on the read length of other samples.

### Repeat clustering and annotation, and 5S rDNA graph-clustering analysis

The repeatome of the *Brachypodium* samples under study was analyzed using RepeatExplorer2 (RE2), a computational pipeline that uses similarity graph-based clustering of filtered PE reads for the identification of the composition and proportion of repetitive elements (Novák et al., 2010, 2013, 2020). Two clustering analyses were performed. First, each sample was analyzed independently using RE2 through the Galaxy platform (https://repeatexplorer-elixir.cerit-sc.cz; Galaxy Version 2.3.8.1) following the protocol of Novák et al. (2020). The clustering analysis of individual samples was fed with variable amount of PE reads per sample to achieve the recommended genome coverage (0.01–0.5×) of each taxon (**Supplementary Table S2**). Clustering was conducted employing the default RE2 settings (90% similarity, minimum overlap = 55; cluster size threshold = 0.01 %) discarding organellar clusters. Automated RE2 cluster annotation was used to quantify the clusters and calculate the proportions of repetitive elements in each sample. Sequence data from the three annual *Brachypodium* samples, enriched with organelle sequences, were pre-processed to remove them using the DUK software (Li et al., 2011) with a k-mer size of 24, a cut-off threshold for 2 matched reads, and the respective plastomes of *B. distachyon* Bd21-3 (LT558596.1), *B. stacei* ABR114 (NC_036837.1; Sancho et al., 2018) and *B. hybridum* ABR113 (NC_036836.1; S-plastotype), as references. Pre-processing steps were applied to format the sequences according to the requirements for subsequent analyses. Thus, split_pairs.v0.5 (https://github.com/eead-csic-compbio/split_pairs; Contreras-Moreira et al., 2016) was used to obtain the interleaved paired input format, and seqtk.v.1.3-r117 (https://github.com/lh3/seqtk) to convert fastq to fasta format. Sequences headers were formatted following the specifications required by the downstream analysis. This independent RE2 analysis resulted in the automatic repeat annotation and quantification of the studied repeatomes.

Secondly, the comparative analysis of all the *Brachypodium* samples under study was carried out using the RE2 program installed on our local server (command repex_tarean/seqclust) using the following parameters: /repex_tarean/seqclust -p -l Brachy_clustering.log -c 0 -P 2 -v Brachy_clustering Brachy_RE.fasta -C -tax VIRIDIPLANTAE3.0 -opt ILLUMINA. This comparative clustering analysis was performed employing the same RE2 settings used for the individual analyses. Organelle clusters and/or clusters with missing data were also removed. The resulting clusters were used for subsequent phylogenetic analysis.

Clustering graph analysis of the 5S data was performed with the Tandem Repeat Analyzer (TAREAN) algorithm implemented in RE2 (Novák et al., 2010; Garcia et al., 2020), available in the Galaxy environment, using the same input indicated above for the individualized RE2 analysis (**Table 1**; **Supplementary Tables S1**, **S2**). The shapes of the 5S rDNA clusters were characterized using a connected component index parameter (C) and their k-mer score was calculated as the sum of the frequencies of all k-mers used for consensus sequence reconstruction (Garcia et al., 2020). The graph topologies of the 5S rDNA cluster were visually inspected and classified into three groups of graph (type 1, simple circular-shaped graph; type 2, complex graph with two loops; type 3, complex graph with three loops); in the complex graphs, interconnected loops represent IGS spacers (Garcia et al., 2020). The 5S graphs were inspected to detect potential variation of 5S rDNA loci (5S ribotypic families) and to identify presumable hybrids and allopolyploids.

### Correlations of repeat amounts and genome size variation in *Brachypodium*

To analyze the potential contribution of the different repeat types and the repeatome to the variation in monoploid genome size (1Cx) observed between and within the *Brachypodium* lineages and samples studied, we performed a test search using the data from the individual analysis and the linear regression model analyses (Pearson correlation coefficient) with the *ggscatter* function from the *ggpubr* package (Kassambara, 2023) in R v.4.0.5 (R Core Team, 2021). Estimation of the monoploid genome size (Cx) from the holoploid genome size (C) is not straightforward in *Brachypodium* allopolyploids, since most of them (all except autopolyploid *B. boissieri*) show dysploid subgenomes (Sancho et al., 2022, and unpub. data). However, we have assumed that the arithmetic mean of the number of genomes/subgenomes is the best approach to estimated it (e. g., allotetraploids (4x): Cx = C/2; auto- and allohexaploids (6x): Cx = C/3). The respective contributions of repeats to pairwise differences in genome sizes were estimated following Macas et al. (2015) and Moreno-Aguilar et al. (2022) using absolute amounts (Mbp) of repeats calculated for individual species (**Supplementary Tables S3, S4**). We also tested whether there were significant differences in repeat amount for different repeat families obtained from the individual analysis through Kruskal–Wallis rank tests using the *PMCMRplus* package (Pohlert, 2023) in R.

### Landscape genomic diversity analysis of repeat types in *Brachypodium*

To investigate the levels of conservatism or diversity of the repeat types that contributed most to genome size variation in *Brachypodium* (44 studied samples) we performed a genome landscape search for the global variability of these individual repeat types across the *Brachypodium* genomes following Macas et al. (2015) and Moreno-Aguilar et al. (2022). We pooled the pairwise similarity values of reads, retrieved from the RE2 outputs (hitsort files) of the global comparative analysis (all samples together), for each sample and repeat type in a separate dataset and evaluated their similarities with respect to similarities of reads from the same repeat in all other samples. We then calculated the ratios of intraspecific versus interspecific similarity matches (Hs/Ho hit ratios), considering that conservative sequence repeats will produce similarity hits with approximately the same frequency for Hs and Ho, while diversified sequence repeats will generate similarity hits with different frequencies (Macas et al., 2015). We also calculated similarity hit ratios for the 5S tandem-repeat rDNA to compare its gene-conserved vs IGS-variable Hs/Ho ratios with those obtained from the other repeat elements analyzed (Moreno-Aguilar et al., 2022).

### Repeatome phylogenomic network of *Brachypodium*

We performed phylogenomic analyses with the repeat data obtained from the comparative clustering of the *Brachypodium* repetitive elements. The repeatome super-network was inferred following the steps described by Vitales et al. (2020a). The most abundant repeats (top 342 clusters), defined as possessing more than 0.01% of the total input reads in the dataset, were employed as the starting data set for phylogenetic analyses. Organelle clusters (plastid, mitochondrial) were removed prior to the phylogenetic inference. For each cluster, the initial data set consisted of the matrices of the observed/expected number of edges between species, which is a measure of the pairwise similarity between the species’ reads. These matrices were extracted from the RE results folder using a custom Perl script (Moreno-Aguilar et al., 2022). Incomplete matrices lacking pairwise similarity or with zero values were excluded. These similarity matrices were transformed into distance matrices by calculating the inverse of the values. The NJ function from the *ape v.5.4-1* package (Paradis and Schliep, 2019) in R was used to build the neighbor-joining trees for each of the 55 surviving clusters. The super-network was constructed using the default parameters in SplitsTree4 v.4.17.0 (Huson and Bryant, 2006). Potential phylogenetic information from the repeatome data set was assessed by topological comparisons of the repeatome network with *Brachypodium* phylogenomic trees retrieved from transcriptome (Sancho et al., 2022) and plastome and nuclear 35S and 5S rDNA data (Díaz-Pérez et al., 2018, and unpub. data).

## Results

### Characterization and quantification of the *Brachypodium* repeatome

**Figure 1:**
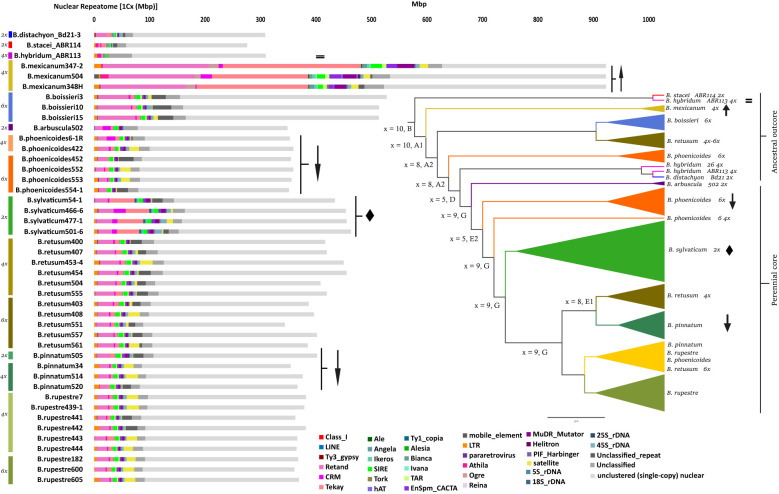
Histograms of repeat contents per monoploid genome (1Cx) retrieved from the individual RepeatExplorer2 analyses of the studied *Brachypodium* samples. Colour codes for species - cytotypes are indicated in the left part of the figure. Colour codes for repeat subfamilies are indicated in the chart. The inset shows a summarized Astral species tree (supertree of whole plastome and nuclear 35S and 5S rDNA trees) of the studied samples (Decena, Sancho, Inda, Perez-Collazos, and Catalan, unpub. data). Karyotypes and subgenomes correspond to those retrieved in Sancho et al. (2022) and unpub. data; inferred karyotypes of ancestors are highlighted in bold. Scale bar: number of mutations per site. Symbols in the histograms and the summarized tree specify examples of the three alternative scenarios of repeatome size variation in response to the polyploid shock hypothesis (arrow up: *B mexicanum*, exacerbated increase of repeatome; equality sign: *B hybridum*, equivalent amount of repeatome to that of diploid progenitor species; arrow down: *B phoenicoides*, *B pinnatum*, considerable reduction of repeatome with increasing ploidy-level) and of potential diploidized paleopolyploidy (diamond: *B sylvaticum*).

The annotated repeats recovered by RE2 in the individual analysis showed remarkable differences in repeat types and contents among the 44 *Brachypodium* samples studied (**Supplementary Tables S3, S4**; **Figure 1**). The proportion of the monoploid genome occupied with repeats ranged from 67.97% (*B. mexicanum*-4x) to 20.77% (*B. stacei*-2x), with a genus-wide average of 28.65% (**Supplementary Tables S3, S4**; **Figure 1**). The amount and 1Cx-percent coverage of repetitive elements varied considerably within both *Brachypodium* polyploids and diploids. Among the polyploids, the highest percentages corresponded to the ancestral outcore perennial tetraploid samples of *B. mexicanum* (56.6-67.9%; mean 60.81%), which exceeded those of all the remaining samples, followed by ancestral outcore perennial hexaploid samples of *B. boissieri* (29.35-32.2%; mean 30.92%), and the lowest to the tetraploid and hexaploid samples of the intermediately evolved *B. retusum* and the recently evolved core perennial *B. pinnatum, B. phoenicoides* and *B. rupestre* (22.5-27.9%), and the tetraploid annual sample of *B. hybridum* (22.05%). Within the intermediately-to-recently-evolved groups, some species showed higher, non-overlapping ranges of repeatome percentages in diploids and low polyploids than in high polyploids [e. g., *B. pinnatum* diploids (26.7%) *vs* tetraploids (22.5-24.9%; mean 23.9%), *B. phoenicoides* tetraploids (25.9-27.9%; mean 26.9%) *vs* hexaploids (22.7-24.3%; mean 23.2%)], while others showed overlapping ranges [e. g., *B. retusum* tetraploids (25.9-27.9; mean 27.2%) *vs* hexaploids (24.9-27.2%; mean 26.1%), *B. rupestre* tetraploids (23.4-25.4%; mean 24.6%) *vs* hexaploids (24.2-24.9%; mean 24.6%)] (**Supplementary Tables S3, S4**; **Figure 1**). Among the diploids, the highest percentages corresponded to the recent core-perennial *B. sylvaticum* samples (32.9-36.1%, mean 34.2%), which notably exceeded those of other core-perennial diploids (*B. pinnatum* 26.7%, *B. arbuscula* 22.54%) and of ancestral outcore diploid annuals (*B. distachyon* 22.75%, *B. stacei* 20.77%). The allotetraploid annual sample of *B. hybridum* showed a 1Cx-percentage coverage of repeatome equivalent to the mean between those of its diploid annual progenitor species *B. stacei* and *B. distachyon* (**Supplementary Tables S3, S4**; **Figure 1**).

LTR-*Gypsy* retrotransposons represented the major repeat fractions in all *Brachypodium* genomes studied, followed by satellite repeats, LTR-*Copia* retrotransposons, and Class II TIR-transposons (**Supplementary Tables S3, S4**; **Figure 1**). The LTR-*Gypsy* Retand (mean 6.75%) and Tekay (3.91%) elements were the most represented repeats in all genomes. Of all elements, Retand repeats covered the highest percentages of genomes in almost all species and similar values in most core-perennial clade samples (16.6-20.7% *B. mexicanum*, 10.5-13.2% *B. boissieri*, 5.2-8.2% *B. retusum*, 4-6.2% core perennials, 6.8% *B. distachyon*, 1.8-0.06% *B. stacei, B. hybridum*; non-significant differences in Kruskal-Wallis tests, **Supplementary Tables S3, S4**). Tekay repeats showed considerable percent differences among species, being highly abundant in the *B. mexicanum* (18.8-27%) and *B. sylvaticum* (8.7-10.6%) genomes (more frequent than the Retand elements), and less abundant in annuals (1-3%) and the remaining core perennial genomes (0.6-3.7%) (significant differences in Kruskal-Wallis tests). LTR-*Copia* SIRE elements (1-2.2%; mean 1.65%) were relatively evenly distributed across *Brachypodium* genomes and showed similar coverage percentages to Tekay elements in most core perennial species (0.6-3.7%). Other types of repeats showed in general proportions <1% in most genomes with some exceptions in those of the *B. mexicanum* samples. Class II-TIR Mutator (0.05-3.4%; mean 0.98%) and CACTA transposons (0.2-2.3%; mean 0.86%) were also evenly distributed across the *Brachypodium* genomes, although the former were more abundant in *B. mexicanum* (Mutator: 2.8%; CACTA: 1.8%) and *B. sylvaticum* (Mutator: 1.64%; CACTA: 1.09%) than in the genomes of other species. Similarly, LTR-*Copia* Ikeros (0.22%) and Angela elements (0.2%) were more frequent in *B. mexicanum* (~0.5%, ~0.7%) and *B. sylvaticum* (~0.38%, ~0.4%), and Angela also in the annual species (0.6-1.5%), than in the remaining *Brachypodium* genomes (<0.3%). LTR-*Copia* TAR elements (0.45%) were present in all genomes except *B. sylvaticum*, while Tork elements (0.18%) were absent in *B. sylvaticum*, most *B. mexicanum* and a few core perennial genomes. LTR-*Gypsy* Ogre elements were found exclusively in the *B. mexicanum* genomes (1.14-1.93%). LTR-*Copia* Bianca (0.17%), Ivana (0.05%), Ale (0.02%) and Alesia (<0.01%), LTR-*Gypsy* Athila (0.13%) and Reina (<0.01%), and Class II Harbinger (0.26%), Helitron (0.02%) and hAT (0.01%) elements were only residually present in a few genomes. Nonspecific tandem satellite repeats (2.23%) were generally well, moderately, or poorly represented in most *Brachypodium* genomes, although their frequencies were unevenly distributed among different groups (**Supplementary Tables S3, S4**). The variation in the satellite fraction between intraspecific samples showing the same cytotype could be due to different factors, such as multiple origins, but also their different dynamics (explosion vs deletion) in separate evolutionary lines, or even incomplete coverage of genomes/subgenomes by the genome scan data. The *B. mexicanum* obese genomes had the highest percentages of genome coverage for most repeat families (**Supplementary Tables S3, S4**; **Figure 1**). Kruskal-Wallis rank tests performed for each of the *Brachypodium* repeat elements found significant differences for the Tekay, Angela, Bianca, TAR, Tork, Helitron, and satellite repeats when examined across all samples (**Supplementary Table S4**).

### Global variability and genomic landscape of the *Brachypodium* repeatome

**Figure 2:**
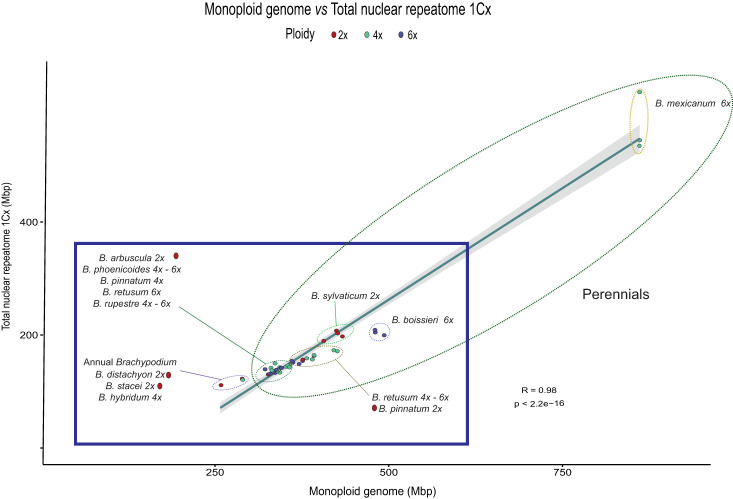
Correlation plot of repeat content and genome size variation (1Cx) for the studied *Brachypodium* samples. Summed abundance values of the most represented repeat types obtained from the individual RepeatExplorer2 analysis. Pearson correlation analysis (R = 0.98, p < 2.2e^-16^). Ellipses with dashed lines encircle the main *Brachypodium* groups. Colour codes for ploidy level are indicated in the chart.

Regression model analysis of repeat content and differences in monoploid genome size between *Brachypodium* samples showed a strong correlation when data from all major repeats were combined (R^2^ = 0.98, p < 2.2E-16), which represents a 49.4% difference in genome size between species (**Table 2**; **Figure 2**). Most repetitive elements (22) presented high correlations. Among them, Retand had the highest correlation values (R^2^ = 0.96, p = 5.41E-30), followed by Ikeros (R^2^ = 0.91, p = 9.07E-24), Tekay (R^2^ = 0.87, p = 7.45E-20), Mutator (R^2^ = 0.84, p = 2,08E-18), Ogre (R^2^ = 0.83, p = 1,79E-17), and others (**Table 2**). The repeat family that accounted for the highest contribution to pairwise differences in genome sizes was Retand (21.7%), followed by Tekay (6.69%), while contributions from the other repeats were <3% (**Table 2**; **Supplementary Figure S2**).

**Table 2:** Pearson linear correlation of repeat abundance with genome size variation (1Cx) in *Brachypodium* and contribution of individual repeats to the genome size differences between species.

Repeat	Correlation to genome size		Abundance in analysed genomes [Mb/1Cx]		Average contribution to pairwise differences in genome sizes [%]
	R^2^	P-value	min.	max	
Retand	**0.955**	**5.41E-30**	0.19	191.01	**21.7**
Tekay	**0.865**	**7.45E-20**	1.97	249.42	**6.69**
SIRE	**0.698**	**1.75E-12**	2.92	20.48	**1.62**
MuDR_Mutator	**0.842**	**2.08E-18**	0.15	31.28	**1.45**
EnSpm_CACTA	**0.765**	**8.92E-15**	0.62	21.22	**1.24**
rDNA	**0.169**	**5.62E-03**	0.19	13.89	**1.12**
TAR	**0.744**	**5.40E-14**	0.00	8.03	**0.551**
CRM	**0.129**	**1.66E-02**	0.00	21.87	**0.516**
Ikeros	**0.912**	**9.07E-24**	0.34	6.09	**0.45**
Angela	**0.344**	**2.90E-05**	0.00	7.01	**0.426**
LTR	**0.106**	**3.13E-02**	0.13	12.92	**0.388**
PIF_Harbinger	**0.13**	**1.61E-02**	0.00	3.23	**0.3**
Bianca	**0.578**	**2.18E-09**	0.00	5.44	**0.265**
Ivana	**0.784**	**1.51E-15**	0.00	2.58	**0.112**
Ogre	**0.825**	**1.79E-17**	0.00	17.81	**0**
Ale	**0.804**	**1.92E-16**	0.00	1.85	**0**
hAT	**0.517**	**3.83E-08**	0.00	3.05	**0**
Athila	**0.298**	**1.25E-04**	0.00	13.38	**0**
mobile_element	**0.298**	**1.26E-04**	0.00	8.58	**0**
Helitron	**0.269**	**3.10E-04**	0.00	3.23	**0**
Reina	**0.213**	**1.62E-03**	0.00	0.18	**0**
satellite	0.0133	4.56E-01	0.03	22.99	1.32
Tork	0.0354	2.21E-01	0.00	3.14	0.13
LINE	0.0503	1.43E-01	0.00	0.87	0.0724
pararetrovirus	0.0635	9.90E-02	0.00	1.11	0.0109
Alesia	0.016	4.13E-01	0.00	0.28	0
Ty3_gypsy	0.016	4.13E-01	0.00	1.39	0
Ty1_copia	0.00277	7.35E-01	0.00	3.27	0
Class_I	0.000885	8.48E-01	0.00	0.46	0
Unclassified	**0.326**	**5.12E-05**	14.19	41.75	**2.86**
Unclassified repeat conflicting	0.004	6.83E-01	0.00	33.65	0.618
All	**0.961**	**3.97E-31**	57.76	627.19	**49.4**

Only the most represented *Brachypodium* repeat types are shown. Significant values are highlighted in bold.

**Table 3:** Genomic pair-end read features of 5S rDNA loci and cluster graph parameters of the studied *Brachypodium* cytotypes.

Taxon-Cytotype	Code	No.Reads in cluster	Genome proportion (%)	Consensus repeat length (bp)	k-mer coverage	Connected component index C	Graph shape (type)
*B.stacei-2x*	Bsta_ABR114	970	0.071	270	0.974	0.997	1
*B.distachyon-2x*	Bdis_Bd21-3	1450	0.095	370	0.844	0.995	1
*B.hybridum-4x*	Bhyb_ABR113	315	0.01	272	0.818	0.8	2
*B.arbuscula-2x*	Barb502	4887	0.28	400	0.801	0.983	1
*B.boissieri-6x*	Bboi3	1153	0.1	271	0.82	0.989	1
	Bboi10	2072	0.16	271	0.823	0.964	2
	Bboi15	1253	0.12	271	0.815	0.974	1
*B.mexicanum-4x*	Bmex347	5483	0.18	273	0.758	0.992	1
	Bmex348H	6625	0.17	260	0.719	0.979	1
	Bmex504	9701	0.26	339	0.767	0.981	1
*B.phoenicoides-4x*	Bpho6-1R	9781	0.44	375	0.825	0.998	2
	Bpho422	2766	0.16	427	0.614	0.996	3
*B.phoenicoides-6x*	Bpho452	2048	0.1	430	0.712	0.976	2
	Bpho552	3282	0.17	376	0.767	0.967	2
	Bpho553	3404	0.15	378	0.585	0.986	2
	Bpho554-1	2356	0.11	376	0.717	0.999	2
*B.pinnatum-2x*	Bpin505	1580	0.079	373	0.926	0.98	1
*B.pinnatum-4x*	Bpin34	1028	0.059	373	0.919	0.989	2
	Bpin514	1425	0.1	373	0.817	0.965	2
	Bpin520	1427	0.072	373	0.929	0.999	2
*B.retusum-4x*	Bret400	1486	0.081	273	0.616	0.99	2
	Bret407	2298	0.12	276	0.669	0.932	3
	Bret453-4	1081	0.069	408	0.762	0.981	3
	Bret454	2938	0.17	373	0.577	0.938	3
	Bret504	2248	0.13	373	0.647	0.944	3
	Bret555	2196	0.13	373	0.614	0.957	3
*B.retusum-6x*	Bret403	2737	0.12	375	0.705	0.946	3
	Bret408	969	0.059	373	0.818	0.971	2
	Bret551	1684	0.094	274	0.542	0.943	3
	Bret557	2692	0.14	430	0.816	0.974	3
	Bret561	1776	0.1	395	0.563	0.97	2
*B.rupestre-4x*	Brup7	1908	0.11	373	0.81	0.971	2
	Brup439-1	1812	0.12	373	0.731	0.973	2
	Brup441	2391	0.13	373	0.86	0.967	1
	Brup442	2481	0.15	388	0.811	0.969	1
	Brup443	1788	0.097	373	0.877	0.977	1
	Brup444	2646	0.15	397	0.912	0.98	1
*B.rupestre-6x*	Brup182	2283	0.12	365	0.814	0.977	2
	Brup600	1314	0.094	376	0.828	0.978	2
	Brup605	1067	0.07	373	0.668	0.993	2
*B.sylvaticum-2x*	Bsyl54-1	4704	0.22	391	0.858	0.997	1
	Bsyl466-6	1672	0.074	392	0.862	0.995	1
	Bsyl477-1	3092	0.14	393	0.815	0.961	1
	Bsyl501-6	2089	0.091	393	0.752	0.956	1

Graph shape types (type 1, simple circular-shaped graph with one loop; types 2 and 3, complex graph with two and three loops, respectively, where the interconnected loops represent IGS spacers).

Global variability analysis of individual repeat types in *Brachypodium* genomes showed different histogram profiles of Hs/Ho hit ratios (**Supplementary Figure S3**). Using the histogram of 5S rDNA sequences as a reference, where a narrow main peak near zero on the log(Hs/Ho) x-axis indicated that the ratios of intraspecific Hs to interspecific Ho hit frequencies were close to one, reflecting hence the high sequence conservation of the 5S genes, while a wide right tail of log(Hs/Ho) values ranging from 0.1 to 3, indicated the high divergence of the 5S rDNA IGS sequences (Moreno-Aguilar et al., 2022), the histograms of the ten analyzed repeats showed contrasting patterns. Although most histograms had overall Gaussian distributions for log(Hs/Ho) hit values, most of them presented main peak values >0.5 and a distribution skewed towards positive values 1-3 (Retand, Mutator, Ale, Ivana, TAR, SIRE, satellite), while the others had main peak values close to zero (Tekay, Ikeros, Ogre, CACTA) but also with tails skewed towards positive x-axis values (**Supplementary Figure S3**). These results suggested greater overall conservatism of Tekay sequences and greater diversification of Retand sequences in the *Brachypodium* genome landscape with respect to these two major repeats types, and similar dynamics for the other minor repeats (**Table 2**).

### The *Brachypodium* repeatome phylogenetic network and 5S rDNA graph-clusters

**Figure 3:**
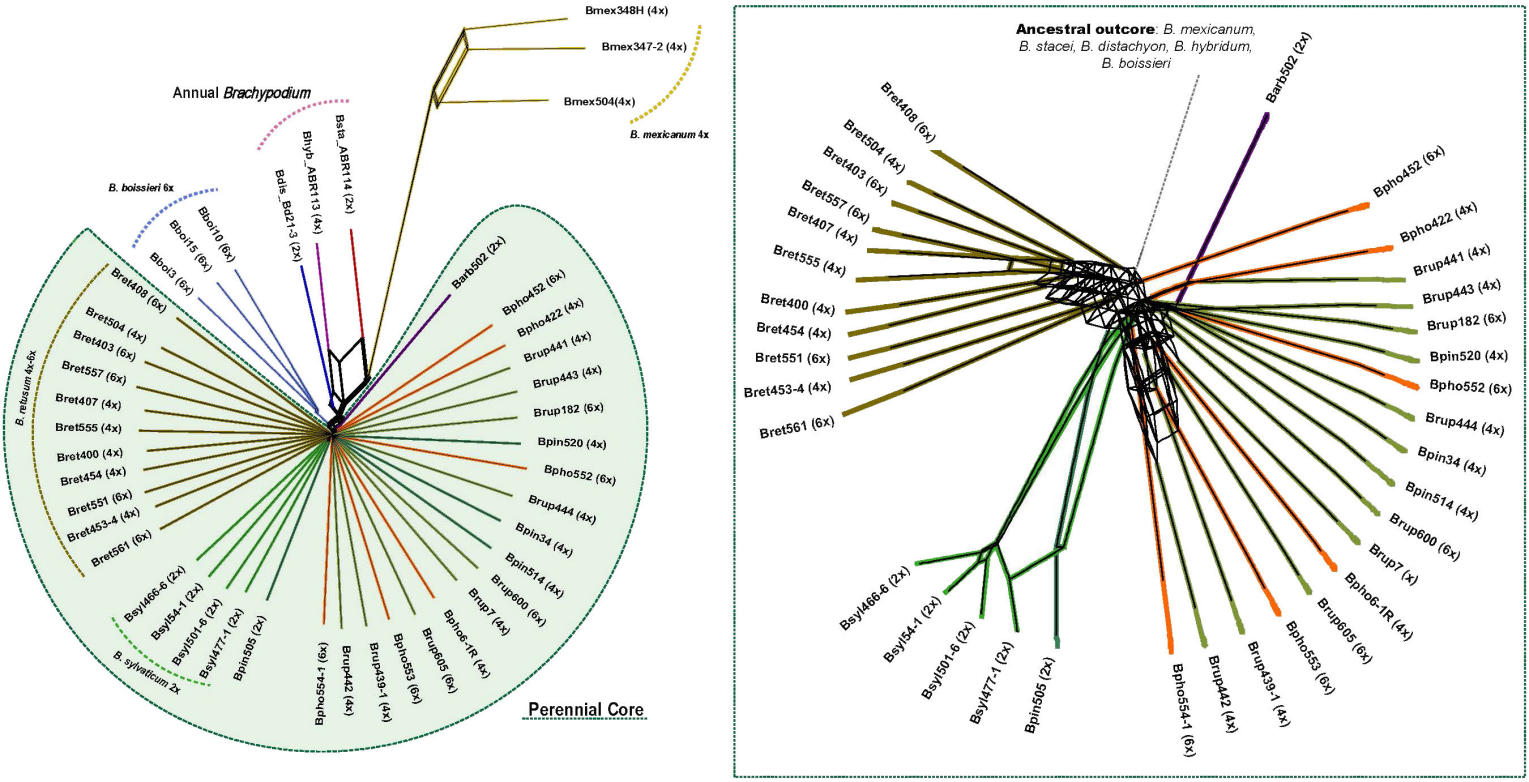
Evolutionary network based on standardized repeat data set obtained from the comparative RepeatExplorer2 analysis of *Brachypodium*. A consensus network was constructed with SplitsTree from distance-based NJ trees computed with transformed similarity matrices to distance matrices by calculating the inverse of the values between samples (see text). Abbreviations of *Brachypodium* species and cytotype samples correspond to those indicated in **Table 1**, and colours codes to those indicated in **Supplementary Figure S1**. The inset shows a detailed view of the *Brachypodium* core-perennial subnetwork.

Comparative analysis of RE2 repeats recovered different types and numbers of shared or species-specific repetitive elements in each *Brachypodium* lineage (**Supplementary Tables S4**, **S5**; **Figure 1**). RE2 annotated different numbers of top clusters in the studied taxa (342 *Brachypodium* clusters, 322 nuclear and 20 organellar; total number of reads in top clusters 2,914,070 (48.1% of total clustered reads) (**Supplementary Table S5**), representing presumably orthologous repeat families from different samples that were clustered due to their high repeat sequence similarity (Macas et al., 2015). We reduced the number of top clusters used to build the NJ trees to 55 clusters after discarding organelle clusters and clusters with NA or zero read values for some samples (**Supplementary Table S5**). The phylogenomic network constructed from the distance-based NJ trees revealed the clear divergences of the ancestral *Brachypodium* outcore lineages and the less resolved relationships of the recent core perennial lineages (**Figure 3**). Among the former group, a *B. mexicanum* cluster was highly isolated from the others, although it was more closely related to the also ancestral *B. stacei* lineage. The allotetraploid *B. hybridum* lineage nested between its two diploid progenitor species *B. stacei* and *B. distachyon* lineages, while the outcore *B. boissieri* cluster was placed close to the *B. distachyon* lineage (**Figure 3**). Within the intricate core-perennial group, a slightly older cluster included most *B. retusum* 4x and 6x samples, the diploid *B. arbuscula* lineage separated from the rest, all diploid *B. sylvaticum* samples grouped into an isolated cluster, and a more recent cluster included the representative samples of the *B. pinnatum* complex taxa (*B. pinnatum* 2x and 4x, *B. rupestre* 4x and 6x, *B. phoenicoides* 4x and 6x) (**Figure 3**).

**Figure 4:**
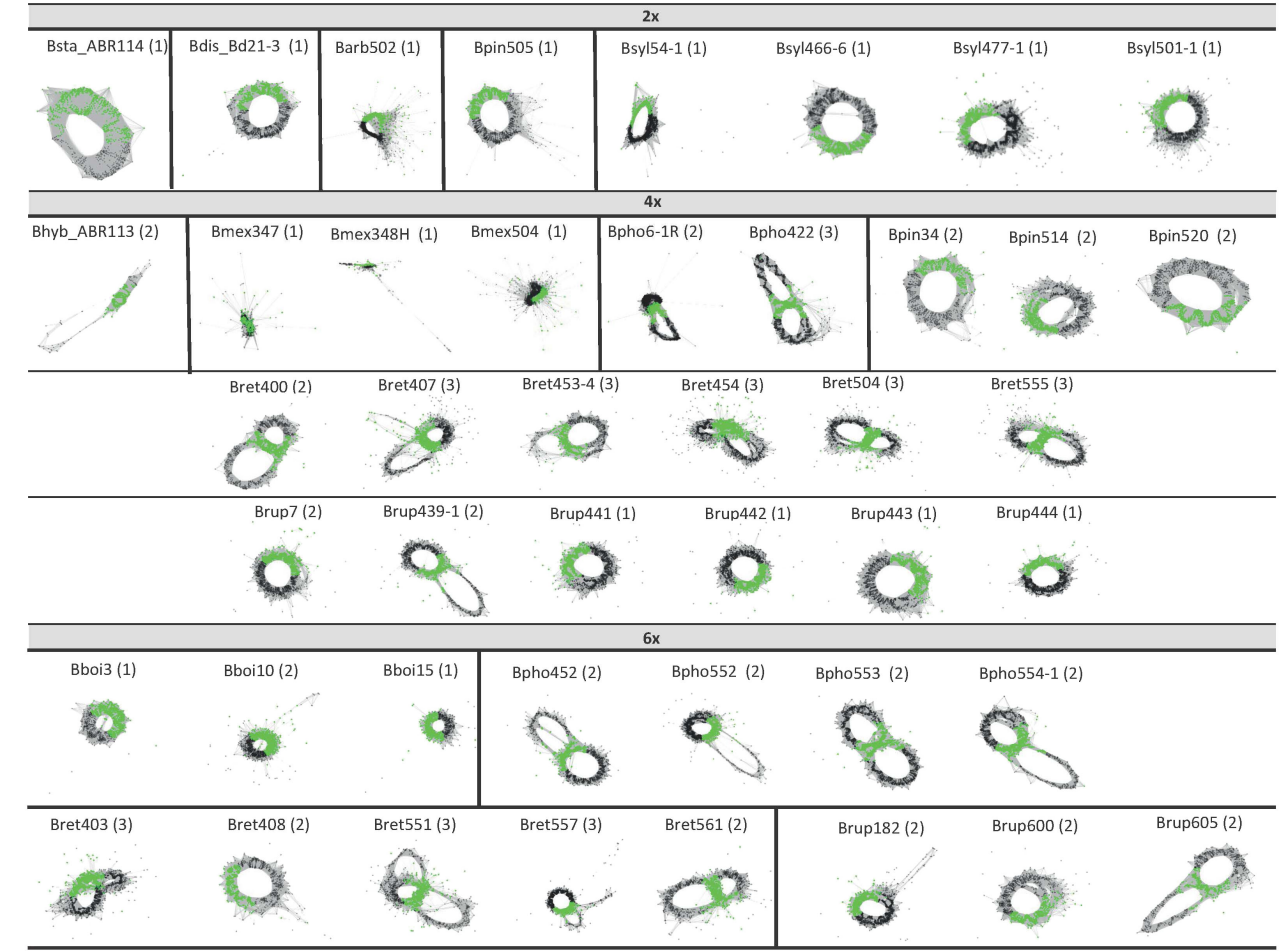
5S clustering graph plots of *Brachypodium* samples generated by the individual RepeatExplorer2 analysis sorted by ploidy level. Diploids (2x) show graph type 1, while some tetraploids and hexaploids show graph types 2 and 3, respectively (see also **Table 3**).

The analysis of the 5S rDNA clusters of the 44 *Brachypodium* samples studied showed different types of simple and complex graphs (**Table 3**; **Figure 4**) that corresponded to short (5S-S) and long (5S-L) 5S sequences. Comparative chromosome barcoding (CCB) analysis using FISH probes has shown that all *Brachypodium* polyploids studied to date, except the autohexaploid *B. boissieri* (subgenomes 8A2 + 8A2 + 8A2), are allopolyploid or autoallopolyploids (Sancho et al., 2022, and unpub. data). These allopolyploids vary from those that have totally different subgenomic karyotypes [*B. hybridum* (4x): 10S + 5D; *B. retusum* (4x): 8A2 + 8E1; *B. phoenicoides* (4x) and *B. rupestre* (4x): 9G + 5E2; *B. retusum* (6x): 8A2 + 8E1 + 5E2] to those with similar segmental karyotypes [*B. mexicanum* (4x): 10A1.1 + 10A1.2], and those with some duplicated subgenomic karyotypes [*B. phoenicoides* (6x) and *B. rupestre* (6x): 9G + 5E2 + 5E2] (Lusinska et al., 2019; Sancho et al. (2022); and unpub. data). Notably, in most cases the retrieved graphs matched the expected types for their respective ploidy levels (**Table 1**; **Supplementary Table S1**), the nature of the polyploidy (auto- *vs* segmental- *vs* allo-polyploidy), and the number and identity of the subgenomes (Sancho et al., 2022; unpub. data). Therefore, the graph topologies of the diploid taxa corresponded to a simple circular type-1 graph that probably represents a single 5S gene family and locus (outcore *B. stacei* and *B. distachyon* and core perennial *B. arbuscula, B. pinnatum* and *B. sylvaticum* 2x samples). In contrast, most allotetraploid samples from *B. hybridum, B. pinnatum*, *B. phoenicoides* and *B. retusum* had complex type-2 graphs showing two IGS loops interconnected by a junction section (coding region of the 5S gene), suggesting they may have two 5S ribotypes. The separation of the two IGS loops was less clear in the graphs of the *B. mexicanum*-4x and *B. pinnatum*-4x samples, while the tetraploid *B. rupestre* samples showed both type-2 (Brup439-1, Brup7) as type-1 graphs (remaining samples) (**Figure 4**). Among the hexaploid taxonomic cytotypes, some *B. retusum*-6x samples (Bret403, Bret551, Bret557) presented complex type-3 graphs with three interconnected IGS loops, indicating that they might have three 5S loci, and the other samples (Bret408, Bret561) type-2 graphs, while all the *B. phoenicoides*-6x and *B. rupestre*-6x samples presented type-2 graphs and all *B. boissieri*-6x samples type-1 graphs (**Figure 4**). Only a few allotetraploid samples (Bpho422, Bret407) showed evidence of more IGS loops than expected (type-3 graphs) (**Figure 4**).

## Discussion

### Delineation of the *Brachypodium* repeatome and its impact on the striking genome size diversification of its lineages

Our comprehensive analysis of the *Brachypodium* repeatome has revealed the composition and frequency of the main repetitive DNA elements across the genome landscape of all its lineages (**Supplementary Table S4**; **Figure 1**). Our data confirm the decisive contribution of the repeatome to the genome size diversification of the studied *Brachypodium* genomes. The repeatome represents a major or considerable percentage of the holoploid genome of the surveyed samples. One of the most noticeable results was the enormous differences in genome sizes, and their correlated repeatome amounts, detected between species and lineages (**Supplementary Tables S3, S4**; **Figures 1**, **2**). For a genus selected as a monocot model system due to the small genome size of its flagship species *B. distachyon* (IBI. The International *Brachypodium* Initiative, 2010; Gordon et al., 2017), differences between the smallest genome sizes found within its annual species (*B. stacei*, holoploid genome 551 Mbp, monoploid genome 275 Mbp), which has the lowest repeatome content (20%), and the largest genome sizes of the slender *B. mexicanum*-4x perennial samples (holoploid genome 3690 Mbp, monoploid genome 922 Mbp), presenting the highest repeatome contents (67.9%), are 6.7-fold and 3.3-fold, respectively (**Table 1**; **Supplementary Tables S1, S3, S4**). Although most of the genomes of the *Brachypodium* species analyzed are small (annual species, monoploid genome 275-309 Mbp) or relatively small (most core-perennial species; ≤352 Mbp) and their respective repeatome percentages are also consistently low (20.7-22.7% annuals; ≤27.9% core perennials), *B. mexicanum*-4x plus the ancestral *B. boissieri*-6x (508 Mbp; 31%) and the recent core-perennial *B. sylvaticum*-2x (450 Mbp; 34%) lineages depart from this trend, and the intermediately evolved *B. retusum*-4x-6x (410 Mbp; 26.7%) also differs slightly from it (**Supplementary Tables S3, S4**; **Figures 1**, **2**).

Surprisingly, the main differences in genome sizes and repeatome amounts have been found between the most ancestral x=10 karyotype lineages, the smallest genomes of *B. stacei* (S karyotype) and the largest genomes of *B. mexicanum* (P and U karyotypes) (**Figure 1**, Sancho et al., 2022). Although the genome (and repeatome) contractions observed in *B. stacei* and in the also ephemeral lineages of *B. distachyon* (intermediately evolved x=5 D karyotype; Sancho et al., 2022) and *B. hybridum* (S+D karyotypes) (**Figure 1**) is a general feature detected in other annual angiosperms (Suda et al., 2015; Pellicer et al., 2018; Hloušková et al., 2019), the gross genomes of the weakly-rhizomatose perennial *B. mexicanum* and the strongly-rhizomatose perennial *B. boissieri* (ancestral x=8 A2 karyotype; Sancho et al., 2022) (**Figure 1**) points toward to a *Brachypodium* common ancestor with an expanded genome that preceded the diversification of its oldest outcore lineages. A similar evolutionary scenario has been hypothesized for the obese-genome ancestor of the *Hesperis* subclade (~1600 Mbp), within the otherwise small-genome Brassicaceae clade, which includes the model dicot *Arabidopsis thaliana* with one of the smallest genome sizes of angiosperms (157 Mbp; Hloušková et al., 2019). Our repeatome data, together with the extremely high collinearity of CCB syntenic blocks detected between the *B. stacei* and *B. mexicanum* chromosomes (Sancho et al., 2022) and high similarity of CCB karyotypes of the P and U subgenomes of *B. mexicanum* (A1.1 and A1.2 in Sancho et al., 2022), suggest that the 3.3-fold differences in the size of their monoploid genomes (for the same number of chromosomes) were caused by expansions of LTR-*Gypsy* retrotransposons in *B. mexicanum* chromosomes (probably coupled with some potential losses in the *B. stacei* chromosomes) and not by WGD (**Supplementary Tables S3, S4**; **Figures 1**, **2**). The inflated genome of the mesopolyploid *B. mexicanum* (10.4-8.6 Ma; Sancho et al., 2022) likely resulted from the proliferation of Tekay (22-27%) and Retand (17-21%) repeat families, and the enrichment in other less abundant elements (Mutator, 2-3%; Angela, 0.7%, Ogre, 1-2%). Interestingly, Ogre retrotransposons, frequent in the genomes of dicotyledonous legumes (Macas et al., 2015) and also common in the genomes of Brassicaceae (Hloušková et al., 2019), were only residually present in some genomes of Loliinae grasses (Moreno-Aguilar et al., 2022), and have been found exclusively in *B. mexicanum* within our low-pass genomic survey of the genus (**Supplementary Tables S3, S1**; **Figure 1**). The relatively large genome of the also ancestral *B. boissieri* (5.4-3.7 Ma, Sancho et al., 2022) was probably the result of the burst of Retand retrotransposons (10-13%), which were also predominant but less enriched in the genomes of the intermediately evolved strong-rhizomatous perennial *B. retusum* (5-8%). The overall decrease in the amounts of Retand (≤ 6%) and other repetitive elements in the genomes of the core-perennial diploids and their derived neopolyploid lineages (**Table 2**; **Supplementary Table S3**; **Figure 1**) was likely a consequence of post-WGD diploidizations and genome downsizings due to the removal of the excess of repeats (Michael, 2014; Hloušková et al., 2019).

The large genome reductions observed in annual *Brachypodium* species of ancestral origin (**Supplementary Tables S3, S4**; **Figure 1**) could also be related to the transition in life form. Evidence suggests that annuality has evolved convergently from perenniality in different lineages of flowering plants, and that it could have been facilitated by evolutionary precursors (correlated developmental, physiological, and genomic traits) in the temperate pooid grasses, which also include *Brachypodium* (Hjertaas et al., 2023). It has also been demonstrated that plants with small genomes can grow in more diverse habitats and tend to be annuals, while those with large genomes are restricted to narrow ecological niches and are perennials (Suda et al., 2015; Pellicer et al., 2018). Although the annual *Brachypodium* species share similar mesic and arid habitats and ranges as other Mediterranean perennial relatives (Catalan et al., 2016), they show shorter generation times and therefore greater dispersal ability and long-distance colonization of new niches and continents than perennials (Scholthof et al., 2018), likely facilitated by their extremely reduced genomes (**Supplementary Tables S3, S4**; **Figure 1**). The large dysploid reduction from the x=10 ancestral S karyotype of *B. stacei* to the x=5 D intermediate karyotype of *B. distachyon* resulted from four nested chromosome fusions; however, the high collinearity of the two genomes was corroborated by their almost similar genomic sizes (Gordon et al., 2020). Our repeatome analysis further support the analogous genomic coverage of repeatome in these diploids (*B. stacei* 20.7%; *B. distachyon* 22.7%), with the differences caused by a possible recent proliferation of Retand elements in the youngest lineage (*B. stacei* 1.2%; *B. distachyon* 6.8%) while the Tekay and Angela elements were slightly higher in the oldest lineage (*B. stacei* 3.3% and 1.5% *vs B. distachyon* 2.6% and 0.9%) (**Supplementary Tables S3, S4**; **Figure 1**). Our data reinforce the findings of Stritt et al. (2020) on the main contribution of Retand elements to the variation of genome sizes among *B. distachyon* accessions, extending it to the entire genus level. These authors also postulated a high dynamic activity and source of intraspecies polymorphisms of very young Angela elements in the *B. distachyon* genome landscape; however, our Hs/Ho ratios indicated a greater diversification of Retand and, to a lesser extent, Tekay sequences in *Brachypodium* genomes (**Supplementary Figure S3**), likely due to the low contribution of the Angela repeats to the genome landscape at the genus level (**Supplementary Table S3**; **Table 3**). Our analysis has also confirmed the low repeatome content of the annual allotetraploid *B. hybridum* (karyotype x=10S + 5D), which showed a balanced percentage (22%) between those of its diploid progenitor species (**Supplementary Tables S3, S4**).

The striking large repeatome coverage of the recently evolved *B. sylvaticum* core-perennial diploid genome (34.2%) relative to other diploid (24.7%) and polyploid (<26%) core-perennial genomes sharing the same recently evolved karyotype x=9 (Sancho et al., 2022) does not correlate with parallel differences in 1Cx genome sizes, which have similar values for *B. sylvaticum*-2x (456 Mbp) as for *B. pinnatum*-2x (401 Mbp) and other core-perennial 2x-4x-6x cytotypes (349-382 Mbp) (**Table 1**, **Supplementary Tables S1, S3, S4**). This unexpected result could be a consequence of a relatively recent polyploidization and subsequent diploidization of the wester lineage of *B. sylvaticum* from the Late Pliocene - Early Pleistocene (2.78 – 2.17 Ma; **Figure 1**; Catalán et al., 2023). *B. sylvaticum* samples showed a proliferation of Tekay retrotransposons (10%) compared to the other core perennial lineages (1-2%), and also higher proportions of Mutator, Ikeros and Angela elements (**Supplementary Tables S3, S4**). This finding, together with other cytogenetic characteristics, such as a greater number of 25 rDNA loci than expected for a diploid (4-6; Wolny and Hasterok, 2009; Breda et al., 2012; Garcia et al., 2017) would suggest a probable post-polyploid diploidized origin. High repeatome coverages were also found in diploidized paleo-polyploids of Loliinae grasses (Moreno-Aguilar et al., 2022).

### Alternative evolutionary responses to the polyploid genome shock hypothesis by different *Brachypodium* allopolyploids

Our study has shown that expansions and contractions in the repeatome are responsible for the three contrasting responses to the PGSH in different allopolyploid *Brachypodium* lineages and that each response was caused by different biological, cytological and temporal scenarios.

Therefore, the exacerbated genome expansion of the old allotetraploid *B. mexicanum* was not a consequence of WGD *per se* but more likely of proliferations of Tekay and Retand retrotransposons and other repetitive DNA elements in the genomes of its progenitor species (**Supplementary Tables S3, S4**; **Figure 1**). TE annotations in the reference genome of this species (Bmex347; Sancho et al., unpub. data) indicated that amplifications of the LTR and other transposable elements were not only limited to (peri)centromeric regions but also to telomeres and chromosome arms. Since TEs could intersperse with coding regions (Hloušková et al., 2019), this distribution would support amplifications of the entire *B. mexicanum* chromosomes through its x=10 karyotype. It is still surprising why the ancestral genome of *B. mexicanum* has the propensity to tolerate or benefit from such repeatome bloating and subsequent genome expansions, which were probably inherited from the common ancestor but not eliminated over time. A plausible explanation could be that the increase in the length of the chromosome arms was compensated by an increase in centromere size and copy number of centromeric tandem repeats that would guarantee correct segregation of obese chromosomes during cell division as it is observed in other grasses (Zhang and Dawe, 2012).

In contrast, the additive repeatome pattern exhibited by the annual allotetraploid *B. hybridum* relative to those of its genome-reduced diploid progenitor species (**Supplementary Tables S3, S4**; **Figure 1**) is a likely response to post-WGD stabilized genome evolution. The three detected independent recurrent origins of this young neopolyploid, spanning the last 1.4 Mya to 20 Kya, ended in the same phenotypic allotetraploid that showed no evidence of homeologous recombination, subgenomic dominance or pronounced TE activations (Gordon et al., 2020; Scarlett et al., 2023; Mu et al., 2023a). It was probably caused by the high evolutionary and structural divergence of the *B. stacei* and *B. distachyon* progenitor genomes (karyotypes x=10S and x=5D) which probably favored the non-recombinant integrity of the resulting subgenomes in the hybrid and the immediate creation of the amphidiploids (Mu et al., 2023a).

The observed reductions in repeatome and genome size with increasing level of ploidy in the recently evolved core-perennial *B. pinnatum* and *B. phoenicoides* lineages (**Supplementary Tables S3, S4**; **Figure 1**) are likely the result of loss of repeats through recombination that resulted in the repeatome contraction (Michael, 2014). Although the polyploid cytotypes of *B. phoenicoides* share a recent subgenome with a *B. pinnatum*-type diploid karyotype (x=9), a second intermediately subgenome with a reduced karyotype (x=5E2) is present once in the allotetraploids (x=9G + 5E2) and twice in the allohexaploids (x=9G + 5E2 + 5E2) (Sancho et al., 2022; unpub. data), thus favoring more frequent recombination between identical or very similar subgenomes and therefore more potential repeatome losses in high polyploids. Parallel to the case of the highly hybridogenic high-polyploid Loliinae lineages, which experienced large genomic rearrangements causing massive repeatome and genome contractions (Moreno-Aguilar et al., 2022), the *B. pinnatum* and *B. phoenicoides* lineages of the core-perennial clade also showed high rates of interspecific hybridization (Khan and Stace, 1999), thus favoring reductions in their repetitive elements and genomes.

### Repeatome-based phylogenomics and concordance between 5S rDNA graphs and *Brachypodium* (sub)genomes

As in previous angiosperms studies (Dodsworth et al., 2015; McCann et al., 2018, 2020; Vitales et al., 2020a; Herklotz et al., 2021; Moreno-Aguilar et al., 2022), shared repeat clusters retrieved from the *Brachypodium* RE2 comparative analysis have demonstrated to contain phylogenetic information for its main lineages (**Figures 1**, **4**; **Supplementary Tables S4, S5**). The topology of the repeatome network constructed from independent distance-based NJ trees (**Figure 3**) is highly congruent with those based on plastome and 35S and 5S rDNA gene trees (**Figure 1**; Díaz-Pérez et al., 2018; and unpub. data) and the *Brachypodium* transcriptome-based subgenomic tree (Sancho et al., 2022). The unrooted network showed the great divergences of the ancestral outcore lineages and the recent separations of the core-perennial lineages (**Figure 3**). The network recovered the high isolation of the *B. mexicanum* lineage from the other lineages; this large divergence resulted from the higher amounts of repeats for the common elements of some repetitive families within the representatives of the genus (**Supplementary Table S5**). The *B. mexicanum* group included two closely related samples from Mexico and a less related sample from Ecuador (**Figure 3**). Although all the *B. mexicanum* samples studied show a similar repeat composition (**Figure 1**), the divergence of the South American Andean sample from the North American Mexican samples coincides with that observed in previous phylogenetic analyses (Díaz-Pérez et al., 2018), indicating the plausible existence of two geographically separated lineages. The closeness of the *B. stacei* lineage to the *B. mexicanum* cluster supports the shared ancestry of the two x=10 karyotypes, and the intermediate location of *B. hybridum* between its two progenitor lineages reinforces its additive pattern. Interestingly, the ancestral *B. boissieri* cluster was resolved as closer to the *B. distachyon* lineage than to the more ancestral *B. mexicanum* and *B. stacei* lineages, matching the relationships recovered in the plastome tree but diverging from that of the nuclear transcriptome trees (**Figure 3**; Sancho et al., 2022). Therefore, the repeatome data also support the additional contribution of a more recently evolved ancestor to the nuclear genome of this putative ancestral autohexaploid species (karyotype x=8A2). Within the recently evolved core-perennial group, the earlier divergence of the *B. retusum* cluster from the rest supports its intermediate evolutionary position between the outcore *B. boissieri* and core-perennial lineages. However, its greater proximity to the core-perennial group suggests a mixed pattern of repeats from ancestral (x = 8A2) and more recent progenitors (x = 8E1 + 5E2) or a convergent evolution towards recent core-perennial progenitor repeats (**Figure 3**), in parallel with plastome- and transcriptome-based findings (Sancho et al., 2022). The respective divergences of the *B. arbuscula* and *B. sylvaticum* lineages from the rest, and the closeness of the taxa and cytotypes of the *B. pinnatum* complex (*B. pinnatum*, *B. rupestre*, *B. phoenicoides*) were probably the result of their specific repeat compositions, particularly those of the highly differentiated *B. sylvaticum* group, and coincided with those recovered from plastomes and rDNA genes (**Figures 1**, **3**; Díaz-Pérez et al., 2018; Sancho et al., 2022; and unp. data).

The 5S rDNA graph topologies (**Table 3**; **Figure 4**) showed a great match with the number and nature of the genomes and subgenomes of the *Brachypodium* samples studied (**Figure 1**; Sancho et al., 2022; unp. data), corroborating their value to uncover ploidy levels, ancient 5S families, and known and orphan subgenomes in angiosperms (Garcia et al., 2020; Vozárová et al., 2021; Moreno-Aguilar et al., 2022). The single type-1 graphs of diploids corresponded to their respective extant monoploid genomes [*B. stacei*, x = 10S; *B. distachyon*, x = 5D; *B. arbuscula, B. pinnatum*, *B. sylvaticum*, x = 9G], type-2 graphs represented the two different subgenomes of allotetraploids [*B. hybridum*, x = 10S + 5D (both extant); *B. phoenicoides*, x = 9G (extant) + 5E2 (orphan); *B. retusum*, x = 8A2 + 8E1 (both orphan)], and type-3 graphs to the three different subgenomes of some allohexaploids [*B. retusum*, x = 8A2 + 8E1 + 5E2 (all orphan)] (**Figure 1**; unpub. data). Interestingly, the poorly resolved type-2 graphs of *B. mexicanum*-4x may indicate that they correspond to close 5S ribotypic families, which coincides with the two similar P and U x=10 orphan subgenomes of this putative ancestral segmental allotetraploid. The type-2 graphs of the *B. phoenicoides*-6x and *B. rupestre*-6x samples would correspond to their two different ribotypes and subgenomes x = 9G (extant) and x = 5E2 (orphan) plus a duplicated x = 5E2 copy (orphan) in these auto-/allohexaploids, and the type-1 graph of *B. boissieri*-6x samples to the triplicated x = 8A2 subgenomes of this ancestral autohexaploid (**Figures 1**, **4**; Sancho et al., 2022; unpub. data). The maintenance of 5S rDNA loci in high allopolyploid *Brachypodium* species is consistent with their conserved patterns in other angiosperm allopolyploids (Garcia et al., 2017). The few cases of fewer 5S graph loops than expected, according to ploidy level and distinct subgenomes (*B. rupestre*-6x; type-1 graphs), could be due to convergent evolution or, more likely, to failure of the low-pass genome sequencing in the detection of the different 5S IGS sequences. In contrast, the few cases of more 5S graph loops than expected (allotetraploids Bpho422 and Bret407; type-3 graphs) could be a consequence of intragenomic IGS heterogeneity of any of the 5S loci (Garcia et al., 2020).

## Conclusions

A genus-wide analysis of the repetitive elements in the genomes of model *Brachypodium* grasses has uncovered three alternative evolutionary scenarios to the PGSH (expansion, stasis, and contractions of repeatome). None of them are related to WGD but instead reflect parental legacies (*B. mexicanum, B. hybridum*) or contraction through recombination in highly hybridogenous polyploids (*B. pinnatum*, *B. phoenicoides*). The model perennial species *B. sylvaticum* may be a diploidized ancestral polyploid. The 5S rDNA graphs describe the types and copies of genomes present in *Brachypodium* species and cytotypes.

## Data availability statement

The datasets presented in this study can be found in the online repository https://github.com/Bioflora/Brachypodium_Repeatome. The accession number(s) can be found in the article/**Supplementary Material**.

## Author contributions

MD: Data curation, Formal analysis, Investigation, Methodology, Visualization, Writing – review & editing. RS: Data curation, Formal analysis, Investigation, Methodology, Software, Supervision, Validation, Writing – original draft, Writing – review & editing. LI: Data curation, Formal analysis, Investigation, Methodology, Visualization, Writing – review & editing. EP-C: Data curation, Formal analysis, Investigation, Methodology, Visualization, Writing – review & editing. PC: Conceptualization, Formal analysis, Funding acquisition, Investigation, Methodology, Project administration, Resources, Supervision, Validation, Visualization, Writing – original draft, Writing – review & editing.

## Supplementary material

Supplementary Figure 1 Geographical distribution of the studied 44 *Brachypodium* samples. (see **Table 1**, **Supplementary Table S1**). Colour codes for taxa and symbol codes for ploidy level (diploid: circle, tetraploid: triangle, hexaploid: square) are indicated in the corresponding charts. **(A)** *B. mexicanum*. **(B)**. *B. arbuscula*, *B. boissieri*, *B. distachyon*, *B. hybridum*, *B. rupestre*, *B. stacei*. **(C)**. *B. phoenicoides*, *B. pinnatum*, *B. retusum*, *B. sylvaticum*.

Supplementary Figure 2 Correlation plots of repeat content and genome size variation (1Cx) for the studied *Brachypodium* samples. Individual plots for the most represented repeat types found across the *Brachypodium* species and cytotype samples (see **Table 2**, **Supplementary Table S3**, **Figure 2**). Colour codes for species –cytotypes are indicated in the chart.

Supplementary Figure 3 Global variability of the main repeat types and their sequence conservation across the *Brachypodium* genomes. Histograms showing distributions of read similarity (Hs/Ho) hit ratios [frequencies of read similarity hits to reads from the same species (Hs) or to reads from all other species (Ho) (log scale, x-axis) and the number of reads (y-axis)]. Hs/Ho ratios close to one (0 on the logarithmic scale) indicate sequence conservatism while larger values indicate sequence diversification.
