# Supplementary material for: Expansions and contractions of repetitive DNA elements reveal contrasting evolutionary responses to the polyploid genome shock hypothesis in Brachypodium model grasses
Source: Front Plant Sci. 2024 Jul 10;15:1419255. doi: 10.3389/fpls.2024.1419255 (PMC11266827; doi:10.3389/fpls.2024.1419255)
Supplement: Supplementary Figure 1 — Geographical distribution of the studied 44 Brachypodium samples. (see Table 1 , Supplementary Table S1 ). Colour codes for taxa and symbol codes for ploidy level (diploid: circle, tetraploid: triangle, hexaploid: square) are indicated in the corresponding charts. (A) B. mexicanum. (B). B. arbuscula, B. boissieri, B. distachyon, B. hybridum, B. rupestre, B. stacei. (C). B. phoenicoides, B. pinnatum, B. retusum, B. sylvaticum. [file DataSheet_1.zip › Data Sheet 1/Supplementary Table S1.pdf]

Supplementary Table S1. Supplementary information on samples included in the repeatome analysis *of Brachypodium*. Taxon authorship, sample's code, chromosome number (2n), genome size (2C/pg), inferred ploidy level (nx), detailed localities and vouchers, and sources of cytogenetic and genomic data [Chromosome number (2n), Genome size (2C), Repeat content (RC)]. Ploidy levels were inferred from chromosome countings and genome sizes (this study and previous records). Asterisks indicate *B. mexicanum* samples Bmex348H and Bmex504 that were inferred to have the same 2n and 2C values and ploidy level as their conspecific reference genome sample Bmex347 based on similar repeat content (see also Figure 1).

Taxon	Code	Chromosome number (2n)	Genome Size (2C/pg)	Ploidy (nx)	Locality	Data source
B.distachyon (L.) P. Beauv.	Bdis_Bd21-3	10	0.631±0	2x	Iraq: Salakudin (SRR3944701)	2n, 2C: Wolny and Hasterok, 2009; Catalán et al., 2012; Gordon et al., 2020. RC: this study
B.stacei Catalán, Joch. Müll., L.A.J. Mur & T. Langdon	Bsta_ABR114	20	0.564±0	2x	Spain: Balearic isles. Formentera (SRR423681)	2n, 2C: Catalán et al., 2012; Gordon et al. 2020; Wolny and Hasterok. 2009. RC: this study
B.hybridum Catalán, Joch. Müll., Hasterok & G. Jenkins	Bhyb_ABR113	30	1.265±0	4x	Portugal: Lisbon (SRR3945056; SRR3945058)	2n, 2C: Catalán et al., 2012. RC: this study
B.arbuscula Gay ex Knoche	Barb502	18	0.713±0.004	2x	Spain: La Gomera	2n, 2C : Sancho et al., 2022. RC: this study
B.boissieri Nyman	Bboi3	48	3.236±0.072	6x	Spain: Granada	2n, 2C : Sancho et al. 2022. RC: this study
	Bboi10	48	3.152±0.04	6x	Spain: Granada	2n, 2C, RC: this study
	Bboi15	48	3.149±0.032	6x	Spain: Granada	2n, 2C, RC: this study
B.mexicanum (Roem. & Schult.) Link	Bmex347-2	40	3.774±0.033	4x	Mexico: Hidalgo	2C : Sancho et al. 2022. 2n: Shi et al., 1993. RC: this study
	Bmex348H*	40*	~3.774±0.033*	4x*	Mexico: Puebla. Herbarium B.	RC: this study
	Bmex504*	40*	~3.774±0.033*	4x*	Ecuador: Loja	RC: this study
B.phoenicoides (L.) P. Beauv. ex Roem. & Schult.	Bpho6-1R	28	1.443±0.019	4x	Spain: Huesca	2n, 2C: Sancho et al., 2022. RC: this study
	Bpho422	28	1.469±0.012	4x	Slovakia: Ružomberok	2C: Sancho et al., 2022. 2n, RC: this study
	Bpho452	38	2.176±0.017	6x	Morocco: Rift Mts	2n, 2C, RC: this study
	Bpho552	38	2.204±0.039	6x	Spain: Cadiz	2n, 2C, RC: this study
	Bpho553	38	2.183±0.013	6x	Spain: Malaga	2n, 2C, RC: this study
	Bpho554-1	38	2.155±0.02	6x	Spain: Granada	2n, 2C: Decena 2023a

B.pinnatum (L.) P. Beauv.	Bpin34	28	1.449±0.018	4x	Great Britain: North Wiltshire	2n, 2C, RC: this study
	Bpin505	18	0.822±0.009	2x	Norway: Oslo. USDA_PI 345964	2n, 2C: Sancho et al., 2022. RC: this study
	Bpin34	28	1.449±0.018	4x	Great Britain: North Wiltshire	2n, 2C, RC: this study
	Bpin514	28	1.537±0.012	4x	Turkey: Samsun. USDA PI 206677	2n, 2C, RC: this study
	Bpin520	28	1.499±0.014	4x	Netherlands: Scherpenzeel	2n, 2C, RC: this study
B.retusum (Pers.) P. Beauv.	Bret400	32	1.704±0.024	4x	Spain: Huesca	2n: Sancho et al., 2022. 2C: Wolny & Hasterok. 2009. RC: this study
	Bret403	42	2.373±0.958	6x	Spain: Huesca	2n, 2C, RC: this study
	Bret407	32	1.715±0.017	4x	Spain: Huesca	2n, 2C, RC: this study
	Bret408	42	2.431±0.033	6x	Spain: Navarra	2n, 2C, RC: this study
	Bret453-4	32*	1.840±0.097	4x	Morocco: Rift Mts.	2n, 2C, RC: this study
	Bret454	32*	1.862±0.196	4x	Morocco: Tazza-Bou Idir	2n, 2C, RC: this study
	Bret504	32	1.669±0.026	4x	France: Vic la Gardiole	2n, 2C, RC: this study
	Bret551	42*	2.109±0.025	6x	Spain: Malaga	2C, 2n, RC: this study
	Bret555	32	1.715±0.017	4x	Spain: Granada	2n, 2C, RC: this study
	Bret557	42	2.464±0.026	6x	Spain: Cadiz	2n, 2C, RC: this study
	Bret561	42	2.362±0.046	6x	Spain: Zaragoza	2n, 2C, RC: this study
B.rupestre (Host) Roem. & Schult.	Brup7	28	1.562±0.016	4x	Russia: Ulitsa Pushkina	2n, 2C, RC: this study
	Brup182	38	2.258±0.026	6x	Croatia: Istria	2n, 2C, RC: this study
	Brup439-1	28	1.55±0.022	4x	Spain: Huesca	2n, 2C, RC: this study
	Brup441	28	1.483±0.008	4x	Spain: Leon	2n, 2C, RC: this study
	Brup442	28	1.56±0.03	4x	Spain: Huesca	2n, 2C, RC: this study
	Brup443	28	1.498±0.012	4x	Spain: Guipuzcoa	2n, 2C, RC: this study
	Brup444	28	1.492±0.021	4x	Spain: Lugo	2n, 2C, RC: this study
	Brup600	38	2.216±0.013	6x	France: Nans les Pins	2n, 2C, RC: this study
	Brup605	38	2.265±0.013	6x	France: Pourrieres	2n, 2C, RC: this study
	Bsyl54-1	18*	0.888±0.008	2x	Morocco: Rif Mountains	2n, 2C, RC: this study

B.sylvaticum (Huds.) P. Beauv.	Bsyl466-6	18*	0.928±0.013	2x	Spain: Huesca	2n, 2C, RC: this study
	Bsyl477-1	18*	0.932±0.017	2x	Spain: Lleida	2n, 2C, RC: this study
	Bsyl501-6	18*	0.947±0.01	2x	France: Roquefort les Pins.	2n, 2C, RC: this study
