# Supplementary material for: Expansions and contractions of repetitive DNA elements reveal contrasting evolutionary responses to the polyploid genome shock hypothesis in Brachypodium model grasses
Source: Front Plant Sci. 2024 Jul 10;15:1419255. doi: 10.3389/fpls.2024.1419255 (PMC11266827; doi:10.3389/fpls.2024.1419255)
Supplement: Supplementary Figure 1 — Geographical distribution of the studied 44 Brachypodium samples. (see Table 1 , Supplementary Table S1 ). Colour codes for taxa and symbol codes for ploidy level (diploid: circle, tetraploid: triangle, hexaploid: square) are indicated in the corresponding charts. (A) B. mexicanum. (B). B. arbuscula, B. boissieri, B. distachyon, B. hybridum, B. rupestre, B. stacei. (C). B. phoenicoides, B. pinnatum, B. retusum, B. sylvaticum. [file DataSheet_1.zip › Data Sheet 1/Supplementary Table S2.pdf]

Supplementary Table S2. Brachypodium samples used in the repetitive DNA analysis, and Proportion of reads in top clusters (%) and 1Cx (Mbp) (from RE2 summary). Accession codes for genome data (ENA/NCBI). Genome skimming paired-end (PE) reads per sample and PE reads selected by RepeatExplorer2 per sample in the comparative analyses. Asterisks indicate *B. distachyon*, *B. stacei* and *B. hybridum* reads retrieved from NCBI accessions (see Table 1 and Supplementary Table S1). Monoploid genome sizes (Cx) were estimated from holoploid genome sizes (C) applying the arithmetic mean of the number of genomes/subgenomes (e. g., diploids (2x): Cx = C/1; allotetraploids (4x): Cx = C/2; auto- and allohexaploids (6x): Cx = C/3).

Taxon	Code	Ploidy	ENA/NCBI Accession	Genome Skiming (reads)	RE2 input Reads (101 bp) 0.5x	RE2 input analysed Reads (101 bp)	Proportion of reads in top clusters (%) and 1Cx (Mbp) (from RE2 summary): Total		Plastid		Nuclear	
							%	1Cx (Mbp)	%	1Cx (Mbp)	%	1Cx (Mbp)
B.distachyon **(L.) P. Beauv.**	Bdis_Bd21-3	2x	SRR4236817	253988409*	1527520	1527520	26	80.23	2.15	6.63	23.85	73.59
B.stacei **Catalán. Joch. Müll.. L.A.J. Mur & T. Langdon**	Bsta_ABR114	2x	SRR3944701	53997876*	1365328	1365328	22	60.68	0.85	2.34	21.15	58.33
B.hybridum **Catalán. Joch. Müll.. Hasterok & G. Jenkins**	Bhyb_ABR113	4x	SRR3945056; SRR3945058	160086982* 175683796*	3062302	3062302	23	71.14	0.66	2.04	22.34	69.10
B.arbuscula **Gay ex Knoche**	Barb502	2x	ERS16317876	24180248	1726024	1726024	29	101.11	5.99	20.88	23.01	80.23
B.boissieri **Nyman**	Bboi3	6x	ERS16317877	25125686	7833684	1127035	33	174.06	3.31	17.46	29.69	156.61
	Bboi10	6x	ERS16317879	43351380	7630336	1296259	35	179.82	3.95	20.29	31.05	159.53
	Bboi15	6x	ERS16317880	24927308	7623074	1049297	34	174.52	1.39	7.13	32.61	167.38
B.mexicanum **(Roem. & Schult.) Link**	Bmex347-2	4x	ERS16317891	23665384	9136070	2988496	68	627.47	0.17	1.57	67.83	625.90
	Bmex348H	4x	ERS16317892	20179262	9136070	3820787	57	525.96	0.31	2.86	56.69	523.10
	Bmex504	4x	ERS16317893	22065424	9136070	3783791	60	553.65	1.89	17.44	58.11	536.21
B.phoenicoides **(L.) P. Beauv. ex Roem. & Schult.**	Bpho6-1R	4x	ERS16317895	25233138	3493202	2211485	27	95.26	0.80	2.82	26.20	92.44
	Bpho422	4x	ERS18928866	27171124	3556144	1721391	28	100.57	0.05	0.18	27.95	100.39
	Bpho452	6x	ERS16317898	40151136	5267644	2021816	26	92.22	1.65	5.85	24.35	86.37
	Bpho552	6x	ERS16317901	47496748	5335426	1946085	24	86.22	1.44	5.17	22.56	81.05
	Bpho553	6x	ERS16317902	25397048	5284590	2315228	29	103.19	5.82	20.71	23.18	82.48
	Bpho554-1	6x	ERS16317903	26581080	5216806	2109699	24	84.30	1.33	4.67	22.67	79.63
B.pinnatum **(L.) P. Beauv.**	Bpin505	2x	ERS16317914	23337386	1989892	1989892	32	128.63	5.59	22.47	26.41	106.16
	Bpin34	4x	ERS16317907	25264450	3507728	1756795	29	102.74	4.71	16.69	24.29	86.05
	Bpin514	4x	ERS16317915	57457528	3720758	1390935	27	101.47	2.22	8.34	24.78	93.12
	Bpin520	4x	ERS16317918	22430312	3628768	1980512	25	91.63	2.69	9.86	22.31	81.77
B.retusum **(Pers.) P. Beauv.**	Bret400	4x	ERS16317920	22364040	4125030	1835553	29	120.82	2.83	11.79	26.17	109.03
	Bret407	4x	ERS16317922	35962418	4151658	1914254	29	121.60	1.57	6.58	27.43	115.02
	Bret453-4	4x	ERS16317924	51216698	4454258	1572982	28	125.97	0.41	1.84	27.59	124.12
	Bret454	4x	ERS16317925	45232264	4507514	1763978	30	136.58	2.36	10.74	27.64	125.83
	Bret504	4x	ERS16317927	55784400	4040302	1674933	31	126.50	4.07	16.61	26.93	109.89
	Bret555	4x	ERS16317929	60431068	4151658	1662812	29	121.60	1.26	5.28	27.74	116.32
	Bret403	6x	ERS16317921	35191392	5744540	2211465	30	116.04	3.75	14.50	26.25	101.53
	Bret408	6x	ERS16317923	52690908	5884946	1643744	26	103.03	1.56	6.18	24.44	96.84
	Bret551	6x	ERS16317928	59678832	5105450	1787407	31	106.57	5.33	18.32	25.67	88.24

	Bret557	6x	ERS16317930	49646712	5964832	1862813	28	112.46	1.59	6.39	26.41	106.07
	Bret561	6x	ERS16317931	28323360	5717910	1734687	29	111.65	1.33	5.12	27.67	106.53
***B.rupestre* (Host) Roem. & Schult.**	Brup7	4x	ERS16317932	30392938	3781278	1695272	28	106.93	2.22	8.48	25.78	98.46
	Brup439-1	4x	ERS16317939	39027346	3752228	1505166	27	102.32	1.59	6.03	25.41	96.30
	Brup441	4x	ERS16317940	31698734	3590034	1832351	25	90.65	1.44	5.22	23.56	85.43
	Brup442	4x	ERS16317941	36842858	3776436	1602794	26	99.17	2.27	8.66	23.73	90.51
	Brup443	4x	ERS16317942	29826044	3626346	1851291	27	98.89	2.11	7.73	24.89	91.16
	Brup444	4x	ERS16317943	21179404	3611822	1788089	26	94.85	1.95	7.11	24.05	87.73
	Brup182	6x	ERS16317938	29045220	5466148	1857595	29	106.74	3.74	13.77	25.26	92.97
	Brup600	6x	ERS16317945	24282712	5364476	1402913	27	97.53	3.08	11.13	23.92	86.40
	Brup605	6x	ERS16317946	22914904	5483094	1525977	26	95.99	1.36	5.02	24.64	90.97
***B.sylvaticum* (Huds.) P. Beauv.**	Bsyl54-1	2x	ERS16317952	25968092	2149664	2149664	35	151.98	2.07	8.99	32.93	142.99
	Bsyl466-6	2x	ERS16317955	27603060	2246496	2246496	37	167.90	1.11	5.04	35.89	162.87
	Bsyl477-1	2x	ERS18928867	28562278	2256178	2256178	37	168.63	2.77	12.62	34.23	156.00
	Bsyl501-6	2x	ERS16317959	21353818	2292490	2292490	35	162.08	1.76	8.15	33.24	153.93
