# Supplementary material for: Expansions and contractions of repetitive DNA elements reveal contrasting evolutionary responses to the polyploid genome shock hypothesis in Brachypodium model grasses
Source: Front Plant Sci. 2024 Jul 10;15:1419255. doi: 10.3389/fpls.2024.1419255 (PMC11266827; doi:10.3389/fpls.2024.1419255)
Supplement: Supplementary Figure 1 — Geographical distribution of the studied 44 Brachypodium samples. (see Table 1 , Supplementary Table S1 ). Colour codes for taxa and symbol codes for ploidy level (diploid: circle, tetraploid: triangle, hexaploid: square) are indicated in the corresponding charts. (A) B. mexicanum. (B). B. arbuscula, B. boissieri, B. distachyon, B. hybridum, B. rupestre, B. stacei. (C). B. phoenicoides, B. pinnatum, B. retusum, B. sylvaticum. [file DataSheet_1.zip › Data Sheet 1/Supplementary Table S3.pdf]

Supplementary Table S3. Genome proportion (percentage) of repeats estimated by RepeatExplorer2 for individual *Brachypodium* samples (estimations per monoploid genome, 1Cx). The sample codes correspond to those indicated in Table 1. Unclassified repeat and Unclassified refer to ambiguous annotations as a result of contradictory evidence and no evidence of the repeat type/class, respectively.

				Class I/Ty1 Copia										Class I/Ty3 gypsy							Class I		Class II/TIR				Class II									
SampleCode	Ploidy	Class I	Class I LTR	Ty1 copia	Ale	Alesia	Angela	Bianca	Ikeros	Ivana	SIRE	TAR	Tork	Ty3 gypsy	Athila	Ogre	Retand	CRM	Tekay	Reina	pararetrovirus	LINE	EnSpm CACTA	hAT	MuDR Mutator	PIF Harbinger	Helitron	mobile element	satellite	45S rDNA	18S rDNA	25S rDNA	5S rDNA	Unclassified repeat	Unclassified	TOTAL (%)
Bdis_Bd21-3	2x	0	0.25	0	0	0.09	0.92	0.34	0.11	0.11	1.17	0.4	0.02	0.45	0	0	6.83	0.01	2.62	0.02	0.11	0.01	0.76	0	0.36	0.02	0	0	0.01	2.15	0	0	0.09	1.3	4.6	22.75
Bsta_ABR114	2x	0	0.97	0	0.03	0	1.53	0.07	0.15	0.04	1.06	0.26	0.01	0	1.69	0	1.18	0.64	3.28	0	0	0.07	0.32	0	0.15	0	0	0.04	0.12	0.49	0	0	0.07	2.74	5.86	20.77
Bhyb_ABR113	4x	0	1.95	0	0.11	0	0.59	0.46	0.16	0	0.95	0.19	0.2	0	0.67	0	0.06	1.19	1.2	0	0.28	0.03	0.2	0.02	0.05	0.02	0	0	0.16	0	0.04	0.01	0.01	0	13.5	22.05
Barb502	2x	0	0.04	0	0	0	0.2	0.25	0.27	0.03	1.88	0.22	0.25	0	0	0	4.02	4.22	1.1	0	0.18	0.17	0.34	0	1.49	0.17	0	0	0.06	0.97	0	0	0.28	0	6.4	22.54
Bboi3	6x	0	1.06	0	0	0	0.18	0.19	0.19	0	1.45	0.5	0.26	0	0	0	10.46	0.57	1.42	0	0.15	0.16	0.55	0	0.4	0.21	0	0	1.15	0.66	0	0	0.1	4.85	4.84	29.35
Bboi10	6x	0	0.98	0	0	0	0.17	0.1	0.16	0	1.22	0.47	0.2	0	0	0	11.77	0.45	1.47	0	0.07	0.14	0.62	0	0.43	0.21	0	0	0.22	1.7	0	0	0.16	6.55	4.13	31.22
Bboi15	6x	0	1.12	0	0	0	0.17	0.12	0.21	0.01	1.23	0.47	0.25	0	0	0	13.19	0.46	1.28	0	0.04	0.16	0.63	0	0.42	0.21	0	0	1.52	0.79	0	0	0.12	5.42	4.37	32.19
Bmex347-2	4x	0	1.4	0	0.2	0	0.72	0.2	0.53	0.2	2.22	0.87	0	0	0.28	1.93	20.7	0.81	27.03	0	0	0.02	2.11	0	3.39	0.03	0.01	0	1.67	0.76	0	0	0.18	0	2.71	67.97
Bmex348H	4x	0	0.98	0	0.16	0	0.76	0.22	0.51	0.16	1.02	0.76	0.34	0	0.09	1.93	16.64	0.2	21.84	0	0.07	0.08	0.97	0.18	2.29	0.12	0.04	0.04	1.43	0.66	0	0	0.17	1.15	3.83	56.64
Bmex504	4x	0	0.3	0	0.2	0	0	0.59	0.66	0.28	1.57	0.85	0	0	1.45	1.14	16.83	2.23	18.75	0.02	0.12	0.05	2.3	0.33	2.63	0.35	0.35	0.93	1.33	0.45	0	0	0.26	0.38	3.47	57.82
Bpho6-1R	4x	0	1.5	0	0.03	0	0.05	0.1	0.17	0.03	1.69	0.69	0.31	0	0	0	4.73	4.41	1.95	0	0.02	0.18	0.84	0	1.01	0.35	0	0	0.46	0	0	0	0.44	0.75	6.22	25.93
Bpho422	4x	0	1.87	0	0	0	0.07	0	0.18	0	1.63	0.72	0.35	0	0	0	5.47	0.86	2.07	0	0	0.09	1.35	0	1.03	0.33	0	0	4.92	1.51	0	0	0.16	0.03	5.24	27.88
Bpho452	6x	0	1.63	0	0.01	0	0.06	0.12	0.13	0.03	1.91	0.46	0.18	0	0	0	4.39	0.03	1.14	0	0.17	0.07	0.71	0.02	0.89	0.28	0	0	0.17	0	0	0	0.1	7.56	4.2	24.26
Bpho552	6x	0	0.4	0	0.02	0	0.06	0.12	0.14	0.04	1.81	0.44	0.18	0	0.11	0	4.19	0.61	1.97	0	0.13	0.14	0.74	0	0.79	0.27	0	0	5.27	0.75	0	0	0.17	0	4.42	22.77
Bpho553	6x	0	1.94	0	0.03	0	0.06	0.11	0.15	0.05	1.57	0.44	0	0	0.01	0	4.17	0.61	0.62	0	0.25	0.22	0.61	0	0.84	0.36	0	0	4.42	1.48	0	0	0.15	0	5.15	23.24
Bpho554-1	6x	0	2.06	0	0.02	0	0.08	0.12	0.14	0	1.79	0.46	0.21	0	0.16	0	3.39	0.03	0.56	0	0.11	0.18	0.75	0	0.87	0.2	0	0	0.47	0	0	0	0.11	5.66	5.34	22.71
Bpin505	2x	0	1.14	0	0.01	0	0.01	0.25	0.18	0.01	1.94	0.58	0.27	0	0.01	0	5.64	0.03	2.47	0	0	0.19	1.21	0	1.19	0.25	0.45	0	0.29	1.4	0	0	0.08	4.5	4.58	26.68
Bpin34	4x	0	2.36	0	0	0	0	0.13	0.2	0	1.59	0.48	0.26	0	0	0	5.3	0.61	0.94	0	0	0.07	0.88	0	0.85	0.31	0	0	4.92	1.14	0	0	0.06	0	4.22	24.32
Bpin514	4x	0	1.8	0	0	0	0.13	0.15	0.21	0	1.71	0.57	0.17	0	0	0	5.48	0.58	1.21	0	0	0	1.04	0	0.89	0.3	0	0	6.05	0.72	0	0	0.1	0	3.8	24.91
Bpin520	4x	0	2.58	0	0.02	0	0.1	0.18	0.19	0	1.89	0.47	0.31	0	0.17	0	3.83	0	1.02	0	0	0.13	1	0	0.9	0.27	0	0	0.26	0.63	0	0	0.07	4.1	4.35	22.47
Bret400	4x	0	1.34	0	0	0	0.17	0.19	0.21	0.06	1.64	0.44	0.24	0	0	0	6.59	0.67	2	0	0.15	0.21	0.63	0	0.7	0.22	0	0	0.29	0	0	0	0.08	4.82	5.25	25.9
Bret407	4x	0	0.97	0	0	0	0.12	0.15	0.19	0.08	1.59	0.44	0.21	0	0	0	7.78	0.5	2.45	0	0.13	0.07	0.76	0.01	0.69	0.38	0	0	1.11	1.18	0	0	0.12	3.38	5.07	27.38
Bret453-4	4x	0	2.07	0	0.01	0	0.14	0.16	0.2	0.08	1.76	0.48	0.23	0	0	0	7.75	0.54	1.77	0	0.18	0.14	0.99	0	0.64	0.25	0	0	5.11	0.89	0	0	0.07	0	4.48	27.94
Bret454	4x	0	1.5	0	0.01	0	0.15	0.18	0.18	0.09	1.62	0.4	0.2	0	0	0	7.33	0.6	1.95	0	0.14	0.04	0.62	0	0.71	0.3	0	0	0.52	0.95	0	0	0.17	4.75	4.77	27.18
Bret504	4x	0	1.28	0	0	0	0.04	0.16	0.2	0.1	1.41	0.43	0.22	0	0	0	7.95	0.54	1.9	0	0.16	0.04	1.11	0	0.66	0.33	0	0	1.18	1.21	0	0	0.13	3.74	4.17	26.96
Bret555	4x	0	0.37	0	0	0	0.16	0.13	0.19	0.09	1.62	0.45	0.19	0	0	0	8.17	0.48	3.01	0	0.15	0.03	0.75	0	0.73	0.34	0	0	0.45	0.94	0	0	0.13	4.77	4.58	27.73

Bret403	6x	0	1.36	0	0.05	0	0.13	0.15	0.19	0.05	1.71	0.48	0.32	0	0	0	7.12	0.61	2.2	0	0.09	0.18	0.61	0	0.76	0.37	0	0	1.35	0	0	0	0.12	3.81	4.73	26.39
Bret408	6x	0	1.39	0	0	0	0	0.16	0.21	0	1.87	0.5	0.26	0	0.04	0	5.27	0.63	2.05	0	0	0.1	0.7	0	0.93	0.27	0	0	5.29	1.1	0	0	0.06	0	4.14	24.97
Bret551	6x	0	0.96	0	0	0	0.07	0.15	0.17	0.09	1.57	0.43	0	0	0	0	7.08	0.6	2	0	0.14	0.03	0.94	0	0.68	0.3	0	0	1.39	1.43	0	0	0.09	3.43	4.24	25.79
Bret557	6x	0	1.44	0	0	0	0.1	0.14	0.2	0.07	2.06	0.44	0.17	0	0.02	0	6.93	0.5	1.31	0	0.12	0.21	0.68	0	0.75	0.33	0	0	0.53	1.08	0	0	0.14	4.47	4.44	26.13
Bret561	6x	0	1.47	0	0.03	0	0.07	0.13	0.2	0.05	1.64	0.49	0.32	0	0	0	7.63	0.63	1.83	0	0.12	0.04	0.66	0	0.71	0.29	0	0	4.81	0	0	0	0.1	1.21	4.8	27.23
Brup7	4x	0	1.53	0	0.05	0	0.04	0.13	0.21	0	1.69	0.57	0.21	0	0	0	5.06	0.57	1.65	0	0	0.04	0.74	0	0.89	0.35	0	0	5.45	1.53	0	0	0.11	0	4.59	25.41
Brup439-1	4x	0	1.54	0	0	0	0	0.14	0.23	0	1.76	0.47	0.26	0	0.03	0	5.09	0.58	1.94	0	0	0.02	0.72	0	0.86	0.23	0	0	5.85	1.5	0	0	0.12	0	3.97	25.31
Brup441	4x	0	1.35	0	0	0	0.07	0.13	0.15	0	1.54	0.53	0.28	0	0	0	4.33	0.63	1.62	0	0.2	0.09	0.55	0	0.75	0.34	0	0	0.34	0.9	0	0	0.13	4.83	4.66	23.42
Brup442	4x	0	2.43	0	0	0	0.05	0.15	0.18	0	1.73	0.46	0.24	0	0.01	0	4.84	0.52	0.98	0	0	0	0.86	0	0.79	0.3	0	0	0.34	0.89	0	0	0.15	5.56	3.73	24.21
Brup443	4x	0	0.33	0	0.01	0	0	0.15	0.19	0.01	1.76	0.47	0.25	0	0.01	0	5.35	0.58	3.66	0	0	0.12	1.02	0	0.98	0.39	0	0	5.18	0.61	0	0	0.1	0	4.04	25.21
Brup444	4x	0	1.27	0	0	0	0.01	0.16	0.17	0	1.71	0.46	0	0	0.01	0	5.08	0.58	2.04	0	0	0.04	0.6	0	0.84	0.26	0	0	4.67	1.21	0	0	0.15	0	4.89	24.15
Brup182	6x	0	1.25	0	0	0	0.07	0.15	0.22	0	1.97	0.6	0.24	0	0.01	0	5.9	0.55	1.78	0	0	0.01	0.79	0	0.95	0.35	0	0	4.45	0.93	0	0	0.12	0	4.59	24.93
Brup600	6x	0	0.42	0	0	0	0	0.13	0.17	0	1.71	0.54	0.2	0	0	0	5.3	0.54	2.31	0	0	0.02	1.12	0	0.78	0.21	0	0	5.52	0.98	0	0	0.09	0	4.2	24.24
Brup605	6x	0	2.19	0	0	0	0.07	0.14	0.19	0	1.78	0.6	0.21	0	0	0	5.32	0.58	0.82	0	0.01	0.01	0.72	0	0.88	0.34	0	0	5.22	0.94	0	0	0.07	0	4.64	24.73
Bsyl54-1	2x	0	0.03	0.72	0	0	0.37	0.19	0.38	0.11	1.82	0	0	0	0.29	0	5.24	0.6	10.55	0	0.06	0.06	1.15	0	1.61	0.35	0	0	0.38	0	0	0	0.22	4.3	4.68	33.11
Bsyl466-6	2x	0	1.35	0.72	0	0	0.35	0.2	0.38	0.08	1.92	0	0	0	0.26	0	5.97	4.82	9.35	0	0.01	0.16	0.97	0.08	1.81	0.29	0.03	0	0.86	1.64	0	0	0.07	0	4.76	36.08
Bsyl477-1	2x	0.1	0.69	0.66	0	0	0.39	0.16	0.33	0.03	1.68	0	0	0	0.11	0	6.23	2.04	10.26	0	0.02	0.02	1.38	0	1.68	0.28	0.02	0	2.46	2.55	0	0	0.14	0	3.49	34.82
Bsyl501-6	2x	0	1.33	0.66	0	0	0.39	0.15	0.3	0.03	1.68	0	0	0	0.25	0	5.31	1.9	8.69	0	0.05	0.06	0.87	0.01	1.46	0.3	0.02	0	0.53	2.91	0	0	0.09	2.03	3.92	32.94
**Mean**		0.00	1.28	0.06	0.02	0.00	0.20	0.17	0.22	0.05	1.65	0.45	0.18	0.01	0.13	0.11	6.75	0.89	3.91	0.00	0.08	0.09	0.86	0.01	0.98	0.26	0.02	0.02	2.23	0.95	0.00	0.00	0.13	2.18	4.73	28.65
