# Supplementary material for: Expansions and contractions of repetitive DNA elements reveal contrasting evolutionary responses to the polyploid genome shock hypothesis in Brachypodium model grasses
Source: Front Plant Sci. 2024 Jul 10;15:1419255. doi: 10.3389/fpls.2024.1419255 (PMC11266827; doi:10.3389/fpls.2024.1419255)
Supplement: Supplementary Figure 1 — Geographical distribution of the studied 44 Brachypodium samples. (see Table 1 , Supplementary Table S1 ). Colour codes for taxa and symbol codes for ploidy level (diploid: circle, tetraploid: triangle, hexaploid: square) are indicated in the corresponding charts. (A) B. mexicanum. (B). B. arbuscula, B. boissieri, B. distachyon, B. hybridum, B. rupestre, B. stacei. (C). B. phoenicoides, B. pinnatum, B. retusum, B. sylvaticum. [file DataSheet_1.zip › Data Sheet 1/Supplementary Table S4.pdf]

Supplementary Table S4. Estimated sizes (Mbp) of repeats elements according to their proportions in the monoploid genome (1Cx). Kruskal-Wallis tests for significant differences in repeat proportions for each repetitive element across the studied samples. Significant values are highlighted in bold. Unclassified repeat and Unclassified refer to ambiguous annotations as a result of contradictory evidence and no evidence of the repeat type/class, respectively.

			Class I/Ty1 Copia										Class I/Ty3 gypsy							Class I		Class II/TIR				Class II										
Taxon Code	Class I	Class I LTR	Ty1 copia	Ale	Alesia	Angela	Bianca	Ikeros	Ivana	SIRE	TAR	Tork	Ty3 gypsy	Athila	Ogre	Retand	CRM	Tekay	Reina	pararetrovirus	LINE	EnSpm CACTA	hAT	MuDR Mutator	PIF Harbinger	Helitron	mobile element	satellite	45S rDNA	18S rDNA	25S rDNA	5S rDNA	Unclassified repeat	Unclassified	TOTAL NUCLEAR	unclustered nuclear
Bdis_Bd21-3	0.00	0.77	0.00	0.00	0.28	2.84	1.05	0.34	0.34	3.61	1.23	0.06	1.39	0.00	0.00	21.07	0.03	8.08	0.06	0.34	0.03	2.35	0.00	1.11	0.06	0.00	0.00	0.03	6.63	0.00	0.00	0.28	4.01	14.19	70.20	238.36
Bsta_ABR114	0.00	2.68	0.00	0.08	0.00	4.22	0.19	0.41	0.11	2.92	0.80	0.03	0.00	4.66	0.00	3.64	1.77	9.05	0.00	0.00	0.19	0.88	0.00	0.41	0.00	0.00	0.11	0.33	1.35	0.00	0.00	0.19	7.56	16.16	57.76	218.04
Bhyb_ABR113	0.00	6.03	0.00	0.34	0.00	1.82	1.42	0.49	0.00	2.94	0.59	0.62	0.00	2.07	0.00	0.19	3.68	3.71	0.00	0.87	0.09	0.62	0.06	0.15	0.06	0.00	0.00	0.49	0.00	0.12	0.03	0.03	0.00	41.75	68.20	241.09
Barb502	0.00	0.14	0.00	0.00	0.00	0.70	0.87	0.94	0.10	6.55	0.77	0.87	0.00	0.00	0.00	14.02	14.71	3.84	0.00	0.63	0.59	1.19	0.00	5.19	0.59	0.00	0.00	0.21	3.38	0.00	0.00	0.98	0.00	22.31	78.59	270.07
Bboi3	0.00	5.59	0.00	0.00	0.00	0.95	1.00	1.00	0.00	7.65	2.64	1.37	0.00	0.00	0.00	55.17	3.01	7.49	0.00	0.79	0.84	2.90	0.00	2.11	1.11	0.00	0.00	6.07	3.48	0.00	0.00	0.53	25.58	25.53	154.81	372.66
Bboi10	0.00	5.04	0.00	0.00	0.00	0.87	0.51	0.82	0.00	6.27	2.41	1.03	0.00	0.00	0.00	60.47	2.31	7.55	0.00	0.36	0.72	3.19	0.00	2.21	1.08	0.00	0.00	1.13	8.73	0.00	0.00	0.82	33.65	21.22	160.40	353.38
Bboi15	0.00	5.75	0.00	0.00	0.00	0.87	0.62	1.08	0.05	6.31	2.41	1.28	0.00	0.00	0.00	67.70	2.36	6.57	0.00	0.21	0.82	3.23	0.00	2.16	1.08	0.00	0.00	7.80	4.05	0.00	0.00	0.62	27.82	22.43	165.23	348.06
Bmex347-2	0.00	12.92	0.00	1.85	0.00	6.64	1.85	4.89	1.85	20.48	8.03	0.00	0.00	2.58	17.81	191.01	7.47	249.42	0.00	0.00	0.18	19.47	0.00	31.28	0.28	0.09	0.00	15.41	7.01	0.00	0.00	1.66	0.00	25.01	627.19	295.55
Bmex348H	0.00	9.04	0.00	1.48	0.00	7.01	2.03	4.71	1.48	9.41	7.01	3.14	0.00	0.83	17.81	153.54	1.85	201.53	0.00	0.65	0.74	8.95	1.66	21.13	1.11	0.37	0.37	13.20	6.09	0.00	0.00	1.57	10.61	35.34	522.64	400.10
Bmex504	0.00	2.77	0.00	1.85	0.00	0.00	5.44	6.09	2.58	14.49	7.84	0.00	0.00	13.38	10.52	155.30	20.58	173.01	0.18	1.11	0.46	21.22	3.05	24.27	3.23	3.23	8.58	12.27	4.15	0.00	0.00	2.40	3.51	32.02	533.53	389.21
Bpho6-1R	0.00	5.29	0.00	0.11	0.00	0.18	0.35	0.60	0.11	5.96	2.43	1.09	0.00	0.00	0.00	16.69	15.56	6.88	0.00	0.07	0.64	2.96	0.00	3.56	1.23	0.00	0.00	1.62	0.00	0.00	0.00	1.55	2.65	21.94	91.48	261.33
Bpho422	0.00	6.72	0.00	0.00	0.00	0.25	0.00	0.65	0.00	5.85	2.59	1.26	0.00	0.00	0.00	19.65	3.09	7.43	0.00	0.00	0.32	4.85	0.00	3.70	1.19	0.00	0.00	17.67	5.42	0.00	0.00	0.57	0.11	18.82	100.14	259.03
Bpho452	0.00	5.78	0.00	0.04	0.00	0.21	0.43	0.46	0.11	6.77	1.63	0.64	0.00	0.00	0.00	15.57	0.11	4.04	0.00	0.60	0.25	2.52	0.07	3.16	0.99	0.00	0.00	0.60	0.00	0.00	0.00	0.35	26.81	14.90	86.05	268.64
Bpho552	0.00	1.44	0.00	0.07	0.00	0.22	0.43	0.50	0.14	6.50	1.58	0.65	0.00	0.40	0.00	15.05	2.19	7.08	0.00	0.47	0.50	2.66	0.00	2.84	0.97	0.00	0.00	18.93	2.69	0.00	0.00	0.61	0.00	15.88	81.80	277.45
Bpho553	0.00	6.90	0.00	0.11	0.00	0.21	0.39	0.53	0.18	5.59	1.57	0.00	0.00	0.04	0.00	14.84	2.17	2.21	0.00	0.89	0.78	2.17	0.00	2.99	1.28	0.00	0.00	15.73	5.27	0.00	0.00	0.53	0.00	18.33	82.69	273.13
Bpho554-1	0.00	7.24	0.00	0.07	0.00	0.28	0.42	0.49	0.00	6.29	1.62	0.74	0.00	0.56	0.00	11.91	0.11	1.97	0.00	0.39	0.63	2.63	0.00	3.06	0.70	0.00	0.00	1.65	0.00	0.00	0.00	0.39	19.88	18.76	79.77	271.49
Bpin505	0.00	4.58	0.00	0.04	0.00	0.04	1.00	0.72	0.04	7.80	2.33	1.09	0.00	0.04	0.00	22.67	0.12	9.93	0.00	0.00	0.76	4.86	0.00	4.78	1.00	1.81	0.00	1.17	5.63	0.00	0.00	0.32	18.09	18.41	107.24	294.72
Bpin34	0.00	8.36	0.00	0.00	0.00	0.00	0.46	0.71	0.00	5.63	1.70	0.92	0.00	0.00	0.00	18.78	2.16	3.33	0.00	0.00	0.25	3.12	0.00	3.01	1.10	0.00	0.00	17.43	4.04	0.00	0.00	0.21	0.00	14.95	86.16	268.12

Bpin514	0.00	6.76	0.00	0.00	0.00	0.49	0.56	0.79	0.00	6.43	2.14	0.64	0.00	0.00	0.00	20.59	2.18	4.55	0.00	0.00	0.00	3.91	0.00	3.34	1.13	0.00	0.00	22.74	2.71	0.00	0.00	0.38	0.00	14.28	93.61	282.19
Bpin520	0.00	9.46	0.00	0.07	0.00	0.37	0.66	0.70	0.00	6.93	1.72	1.14	0.00	0.62	0.00	14.04	0.00	3.74	0.00	0.00	0.48	3.67	0.00	3.30	0.99	0.00	0.00	0.95	2.31	0.00	0.00	0.26	15.03	15.94	82.35	284.15
Bret400	0.00	5.58	0.00	0.00	0.00	0.71	0.79	0.87	0.25	6.83	1.83	1.00	0.00	0.00	0.00	27.46	2.79	8.33	0.00	0.62	0.87	2.62	0.00	2.92	0.92	0.00	0.00	1.21	0.00	0.00	0.00	0.33	20.08	21.87	107.91	308.72
Bret407	0.00	4.07	0.00	0.00	0.00	0.50	0.63	0.80	0.34	6.67	1.84	0.88	0.00	0.00	0.00	32.62	2.10	10.27	0.00	0.55	0.29	3.19	0.04	2.89	1.59	0.00	0.00	4.65	4.95	0.00	0.00	0.50	14.17	21.26	114.81	304.51
Bret453-4	0.00	9.31	0.00	0.04	0.00	0.63	0.72	0.90	0.36	7.92	2.16	1.03	0.00	0.00	0.00	34.87	2.43	7.96	0.00	0.81	0.63	4.45	0.00	2.88	1.12	0.00	0.00	22.99	4.00	0.00	0.00	0.31	0.00	20.15	125.70	324.18
Bret454	0.00	6.83	0.00	0.05	0.00	0.68	0.82	0.82	0.41	7.38	1.82	0.91	0.00	0.00	0.00	33.37	2.73	8.88	0.00	0.64	0.18	2.82	0.00	3.23	1.37	0.00	0.00	2.37	4.32	0.00	0.00	0.77	21.62	21.72	123.74	331.52
Bret504	0.00	5.22	0.00	0.00	0.00	0.16	0.65	0.82	0.41	5.75	1.75	0.90	0.00	0.00	0.00	32.44	2.20	7.75	0.00	0.65	0.16	4.53	0.00	2.69	1.35	0.00	0.00	4.82	4.94	0.00	0.00	0.53	15.26	17.02	110.02	298.05
Bret555	0.00	1.55	0.00	0.00	0.00	0.67	0.55	0.80	0.38	6.79	1.89	0.80	0.00	0.00	0.00	34.26	2.01	12.62	0.00	0.63	0.13	3.14	0.00	3.06	1.43	0.00	0.00	1.89	3.94	0.00	0.00	0.55	20.00	19.20	116.28	303.04
Bret403	0.00	5.26	0.00	0.19	0.00	0.50	0.58	0.73	0.19	6.61	1.86	1.24	0.00	0.00	0.00	27.54	2.36	8.51	0.00	0.35	0.70	2.36	0.00	2.94	1.43	0.00	0.00	5.22	0.00	0.00	0.00	0.46	14.74	18.30	102.08	284.72
Bret408	0.00	5.51	0.00	0.00	0.00	0.00	0.63	0.83	0.00	7.41	1.98	1.03	0.00	0.16	0.00	20.88	2.50	8.12	0.00	0.00	0.40	2.77	0.00	3.69	1.07	0.00	0.00	20.96	4.36	0.00	0.00	0.24	0.00	16.40	98.94	297.31
Bret551	0.00	3.30	0.00	0.00	0.00	0.24	0.52	0.58	0.31	5.40	1.48	0.00	0.00	0.00	0.00	24.34	2.06	6.88	0.00	0.48	0.10	3.23	0.00	2.34	1.03	0.00	0.00	4.78	4.92	0.00	0.00	0.31	11.79	14.58	88.66	255.11
Bret557	0.00	5.78	0.00	0.00	0.00	0.40	0.56	0.80	0.28	8.27	1.77	0.68	0.00	0.08	0.00	27.83	2.01	5.26	0.00	0.48	0.84	2.73	0.00	3.01	1.33	0.00	0.00	2.13	4.34	0.00	0.00	0.56	17.95	17.83	104.95	296.69
Bret561	0.00	5.66	0.00	0.12	0.00	0.27	0.50	0.77	0.19	6.31	1.89	1.23	0.00	0.00	0.00	29.38	2.43	7.05	0.00	0.46	0.15	2.54	0.00	2.73	1.12	0.00	0.00	18.52	0.00	0.00	0.00	0.39	4.66	18.48	104.84	280.17
Brup7	0.00	5.84	0.00	0.19	0.00	0.15	0.50	0.80	0.00	6.45	2.18	0.80	0.00	0.00	0.00	19.32	2.18	6.30	0.00	0.00	0.15	2.83	0.00	3.40	1.34	0.00	0.00	20.81	5.84	0.00	0.00	0.42	0.00	17.53	97.04	284.87
Brup439-1	0.00	5.84	0.00	0.00	0.00	0.00	0.53	0.87	0.00	6.67	1.78	0.99	0.00	0.11	0.00	19.29	2.20	7.35	0.00	0.00	0.08	2.73	0.00	3.26	0.87	0.00	0.00	22.17	5.68	0.00	0.00	0.45	0.00	15.05	95.92	283.06
Brup441	0.00	4.90	0.00	0.00	0.00	0.25	0.47	0.54	0.00	5.58	1.92	1.02	0.00	0.00	0.00	15.70	2.28	5.87	0.00	0.73	0.33	1.99	0.00	2.72	1.23	0.00	0.00	1.23	3.26	0.00	0.00	0.47	17.51	16.90	84.92	277.67
Brup442	0.00	9.27	0.00	0.00	0.00	0.19	0.57	0.69	0.00	6.60	1.75	0.92	0.00	0.04	0.00	18.46	1.98	3.74	0.00	0.00	0.00	3.28	0.00	3.01	1.14	0.00	0.00	1.30	3.39	0.00	0.00	0.57	21.21	14.23	92.34	289.08
Brup443	0.00	1.21	0.00	0.04	0.00	0.00	0.55	0.70	0.04	6.45	1.72	0.92	0.00	0.04	0.00	19.59	2.12	13.41	0.00	0.00	0.44	3.74	0.00	3.59	1.43	0.00	0.00	18.97	2.23	0.00	0.00	0.37	0.00	14.80	92.33	273.93
Brup444	0.00	4.63	0.00	0.00	0.00	0.04	0.58	0.62	0.00	6.24	1.68	0.00	0.00	0.04	0.00	18.53	2.12	7.44	0.00	0.00	0.15	2.19	0.00	3.06	0.95	0.00	0.00	17.04	4.41	0.00	0.00	0.55	0.00	17.84	88.10	276.70
Brup182	0.00	4.60	0.00	0.00	0.00	0.26	0.55	0.81	0.00	7.25	2.21	0.88	0.00	0.04	0.00	21.72	2.02	6.55	0.00	0.00	0.04	2.91	0.00	3.50	1.29	0.00	0.00	16.38	3.42	0.00	0.00	0.44	0.00	16.89	91.76	276.30
Brup600	0.00	1.52	0.00	0.00	0.00	0.00	0.47	0.61	0.00	6.18	1.95	0.72	0.00	0.00	0.00	19.14	1.95	8.34	0.00	0.00	0.07	4.05	0.00	2.82	0.76	0.00	0.00	19.94	3.54	0.00	0.00	0.33	0.00	15.17	87.56	273.65
Brup605	0.00	8.09	0.00	0.00	0.00	0.26	0.52	0.70	0.00	6.57	2.22	0.78	0.00	0.00	0.00	19.64	2.14	3.03	0.00	0.04	0.04	2.66	0.00	3.25	1.26	0.00	0.00	19.27	3.47	0.00	0.00	0.26	0.00	17.13	91.30	277.89
Bsyl54-1	0.00	0.13	3.13	0.00	0.00	1.61	0.83	1.65	0.48	7.90	0.00	0.00	0.00	1.26	0.00	22.75	2.61	45.81	0.00	0.26	0.26	4.99	0.00	6.99	1.52	0.00	0.00	1.65	0.00	0.00	0.00	0.96	18.67	20.32	143.77	290.46
Bsyl466-6	0.00	6.13	3.27	0.00	0.00	1.59	0.91	1.72	0.36	8.71	0.00	0.00	0.00	1.18	0.00	27.09	21.87	42.43	0.00	0.05	0.73	4.40	0.36	8.21	1.32	0.14	0.00	3.90	7.44	0.00	0.00	0.32	0.00	21.60	163.73	290.06
Bsyl477-1	0.46	3.14	3.01	0.00	0.00	1.78	0.73	1.50	0.14	7.66	0.00	0.00	0.00	0.50	0.00	28.39	9.30	46.76	0.00	0.09	0.09	6.29	0.00	7.66	1.28	0.09	0.00	11.21	11.62	0.00	0.00	0.64	0.00	15.91	158.69	297.06
Bsyl501-6	0.00	6.16	3.06	0.00	0.00	1.81	0.69	1.39	0.14	7.78	0.00	0.00	0.00	1.16	0.00	24.59	8.80	40.24	0.00	0.23	0.28	4.03	0.05	6.76	1.39	0.09	0.00	2.45	13.48	0.00	0.00	0.42	9.40	18.15	152.54	310.54

			Class I/Ty1 Copia										Class I/Ty3 gypsy							Class I		Class II/TIR				Class II										
	Class I	Class I LTR	Ty1 copia	Ale	Alesia	Angela	Bianca	Ikeros	Ivana	SIRE	TAR	Tork	Ty3 gypsy	Athila	Ogre	Retand	CRM	Tekay	Reina	pararetrovirus	LINE	EnSpm CACTA	hAT	MuDR Mutator	PIF Harbinger	Helitron	mobile element	satellite	45S rDNA	18S rDNA	25S rDNA	5S rDNA	Unclassified repeat	Unclassified	TOTAL NUCLEAR	unclustered nuclear
**mean**	0.01	5.29	0.28	0.15	0.01	0.93	0.78	1.1	0.26	7.04	2.06	0.76	0.03	0.68	1.05	33.89	3.92	23.87	0.01	0.33	0.37	4.04	0.12	4.87	1.11	0.13	0.21	9.12	4.01	0	0	0.58	9.14	19.47	135.63	292.09
**sd**	0.07	2.7	0.91	0.44	0.04	1.54	0.81	1.18	0.51	2.71	1.67	0.58	0.21	2.14	4.03	38.67	5.01	52.24	0.03	0.32	0.28	3.86	0.52	5.98	0.5	0.55	1.29	8.26	2.91	0.02	0	0.45	10.06	5.55	120.14	36.45
**Kruskall wallis test**	4.5	**7.14**	19.3	1.91	4.5	**7.77**	**8.11**	**2.3**	2.98	1.09	**11.97**	**6.09**	4.5	4.82	3.44	0.25	1.91	**8.39**	1.76	0.89	1.42	3.78	1.52	**6.32**	0.91	**9.06**	1.67	**7.48**	2.94	1.1	1.1	0.78	0.93	0.71	0.66	0.78
**p-value**	0.11	**0.03**	0	0.39	0.11	**0.02**	**0.02**	0.32	0.23	0.58	**0**	**0.05**	0.11	0.09	0.18	0.88	0.38	**0.02**	0.41	0.64	0.49	0.15	0.47	0.04	0.64	**0.01**	0.43	**0.02**	0.23	0.58	0.58	0.68	0.63	0.7	0.72	0.68
