# Supplementary material for: Expansions and contractions of repetitive DNA elements reveal contrasting evolutionary responses to the polyploid genome shock hypothesis in Brachypodium model grasses
Source: Front Plant Sci. 2024 Jul 10;15:1419255. doi: 10.3389/fpls.2024.1419255 (PMC11266827; doi:10.3389/fpls.2024.1419255)
Supplement: Supplementary Figure 1 — Geographical distribution of the studied 44 Brachypodium samples. (see Table 1 , Supplementary Table S1 ). Colour codes for taxa and symbol codes for ploidy level (diploid: circle, tetraploid: triangle, hexaploid: square) are indicated in the corresponding charts. (A) B. mexicanum. (B). B. arbuscula, B. boissieri, B. distachyon, B. hybridum, B. rupestre, B. stacei. (C). B. phoenicoides, B. pinnatum, B. retusum, B. sylvaticum. [file DataSheet_1.zip › Data Sheet 1/Supplementary Table S5.pdf]

Supplementary Table S5. RepeatExplorer2 comparative analysis of *Brachypodium* samples. Repeat content data (annotation and number of clustered reads per cluster and sample) for 55 top clusters (repeat families) used for computing Neighbor-Joining (NJ) phylogenetic trees. CL indicates cluster ID.

CL	Cluster size (reads)	Automatic annotation	Barb502	Bboi10	Bboi15	Bboi3	Bpho422	Bpho452	Bpho552	Bpho553	Bpho554-1	Bpho6-1R	Bpin34	Bpin505	Bpin514	Bpin520	Bret400	Bret403	Bret407	Bret408	Bret453-4	Bret452	Bret504	Bret551	Bret555	Bret557	Bret561	Brup182	Brup439-1	Brup441	Brup442	Brup443	Brup444	Brup600	Brup605	Brup7	Bsyl466-6	Bsyl477-1	Bsyl501-6	Bsyl502-5	Bmex347	Bmex348H	Bmex504	Bdis Bd21-3	Bsta ABR114	Bhyb ABR113
2	46197	All	567	1470	1537	1808	996	1479	1520	1449	1868	1067	917	613	970	1117	1083	1526	989	1675	1076	1106	894	1122	1041	1518	1405	1486	1048	1040	1025	992	1029	1555	1519	1056	490	491	487	514	120	119	130	339	502	1442
30	18908	All	40	95	159	144	141	152	152	138	147	145	105	49	95	153	88	133	107	208	108	87	99	108	108	104	132	121	98	130	89	95	95	147	156	117	124	101	140	128	6533	5011	2445	73	100	208
99	10454	All	104	438	481	563	195	318	275	283	339	162	160	108	197	211	292	342	254	315	287	289	238	307	267	343	334	296	206	168	189	174	174	321	308	183	93	93	84	111	133	130	193	76	122	298
175	4306	All	58	129	161	173	97	125	141	135	138	103	81	51	86	114	108	124	83	132	88	83	81	104	100	136	132	149	103	79	100	88	85	156	131	84	47	35	49	49	41	60	86	40	40	121
179	4241	All	45	182	179	176	88	117	166	135	129	109	74	37	79	91	114	135	96	150	109	92	100	130	106	183	189	100	97	84	79	83	89	130	123	68	56	49	34	36	30	41	48	27	26	30
182	4062	All	22	149	161	190	68	121	107	107	125	92	59	31	81	73	119	124	97	119	100	100	70	99	115	147	132	140	75	65	64	66	71	116	113	83	55	24	38	45	65	65	82	42	34	211
193	3589	All	32	121	137	173	82	104	94	105	118	71	62	37	59	78	90	105	75	108	101	98	98	94	96	110	102	112	78	72	74	61	70	108	109	90	21	31	29	25	31	33	47	46	29	173
214	2901	All	20	130	103	181	38	94	56	66	89	50	60	26	62	69	82	75	82	96	66	70	52	72	54	99	62	97	62	34	63	71	36	93	81	61	37	31	50	40	55	58	67	20	21	70
216	2807	All	23	93	113	123	39	51	61	76	81	61	49	33	56	58	82	66	59	77	97	84	57	57	70	110	82	76	48	54	41	42	48	86	68	53	30	29	25	27	54	60	71	26	61	150
251	1896	All	30	47	53	63	36	64	61	54	53	40	41	22	52	57	34	38	38	66	46	41	49	48	33	72	55	59	47	38	46	39	32	67	62	31	20	25	14	22	18	34	51	15	30	53
45	16447	repeat	127	658	316	230	447	514	328	607	519	212	358	193	216	170	288	488	391	591	250	333	428	632	320	541	570	372	445	254	282	196	357	403	424	509	382	438	550	302	672	464	332	269	53	16
55	15520	repeat	121	855	349	338	436	548	276	581	431	187	321	232	183	163	263	361	317	462	217	339	406	497	293	497	447	394	451	249	209	155	353	345	380	456	280	455	528	314	563	531	354	284	76	23
113	9054	repeat	95	477	172	196	233	295	160	304	282	102	195	118	107	92	179	209	192	278	146	171	220	273	148	304	306	212	239	155	148	90	159	280	188	236	175	260	365	174	322	340	204	178	52	23
149	6297	mobile element	59	174	218	223	117	204	166	194	172	126	130	87	146	169	137	208	95	252	149	135	124	154	153	168	171	242	154	131	149	147	134	191	241	156	114	121	119	142	41	57	66	39	51	71
58	15084	LINE	153	751	707	769	328	433	393	419	491	354	297	148	290	240	399	498	307	422	454	366	345	399	314	562	503	440	262	281	254	306	310	412	462	287	173	156	154	149	215	205	343	102	84	147
174	4356	LINE	28	199	223	236	82	135	111	112	114	96	72	48	83	77	101	161	118	108	119	116	100	122	118	118	151	110	72	73	63	76	77	99	109	80	74	60	42	51	81	94	127	38	41	41
34	18521	LTR	181	433	450	477	329	506	577	546	460	264	309	272	404	349	363	544	295	540	540	478	340	458	409	588	542	516	332	289	379	335	296	473	571	402	222	248	279	238	1193	1008	856	36	46	146
107	9891	LTR	70	382	371	492	246	274	262	279	304	231	173	135	221	260	174	305	209	309	281	242	163	268	246	299	261	331	203	233	192	189	214	300	344	263	134	139	106	169	130	86	178	35	48	140
62	14661	Angela	57	230	255	256	78	74	120	96	93	91	60	51	97	70	118	133	134	98	126	119	122	125	128	150	158	122	71	55	75	60	69	90	119	80	154	147	131	142	3527	2466	3167	162	436	549
68	14059	Angela	41	114	115	160	46	42	48	35	72	22	23	25	43	35	52	71	64	67	44	40	50	57	54	72	43	34	47	26	53	36	36	52	45	48	71	52	51	54	4383	3376	4026	48	80	106
109	9615	Angela	35	247	237	272	74	119	80	69	78	47	32	29	87	47	85	114	95	80	111	78	106	93	112	114	98	94	79	70	86	47	65	84	133	81	107	124	122	142	1448	1712	1916	135	247	384
72	13531	Bianca	226	458	511	569	177	362	316	331	305	174	235	187	248	295	270	340	307	410	246	317	226	305	292	342	378	353	254	246	274	251	248	273	368	220	201	156	161	178	386	450	1045	155	62	423
29	19071	Ikeros	181	567	656	644	257	388	348	355	323	257	292	169	342	347	381	474	400	490	438	395	383	389	341	505	444	495	312	326	365	313	285	432	436	360	325	321	281	306	1326	1336	1716	67	97	206
33	18539	SIRE	212	452	419	514	468	599	579	549	566	362	337	195	414	424	459	602	417	653	462	444	394	541	389	699	632	633	407	381	409	433	307	624	694	348	215	230	194	209	479	340	633	65	37	119
36	17252	SIRE	174	426	476	524	270	544	556	520	520	253	334	156	350	344	418	604	410	606	507	448	375	526	415	647	597	506	387	337	355	342	316	538	534	399	177	144	207	182	444	412	804	53	22	93
38	17142	SIRE	192	477	495	471	387	594	554	513	529	322	315	189	359	312	383	530	407	614	497	433	363	536	365	641	549	529	418	325	374	409	350	513	539	334	153	199	178	215	456	373	592	68	28	62
47	16264	SIRE	154	373	481	487	166	570	518	446	525	217	324	164	328	379	371	573	386	574	513	428	334	443	351	664	537	516	335	320	326	314	325	479	590	368	156	158	202	197	345	416	769	34	20	88
64	14136	SIRE	140	564	621	601	220	476	421	289	356	252	297	327	291	365	298	408	299	439	306	259	295	276	233	433	368	492	313	287	309	297	384	410	458	315	240	181	216	247	151	203	302	77	187	233
76	13109	SIRE	148	629	674	795	151	398	375	242	298	216	234	257	291	343	321	370	301	371	319	282	202	345	300	376	342	363	278	215	250	249	281	388	385	243	128	161	180	217	134	184	296	68	190	319
145	6787	SIRE	62	294	327	380	112	221	153	115	176	123	123	58	121	91	108	121	119	171	122	138	117	133	104	143	144	206	87	121	100	92	83	169	159	93	128	100	84	91	158	204	352	198	100	486
11	26649	TAR	95	893	890	990	645	572	648	601	593	611	444	247	571	404	460	741	451	735	549	506	410	556	498	710	724	845	418	418	452	443	371	739	796	564	296	250	287	299	1808	1697	1955	174	104	189
132	7807	TAR	47	323	285	344	237	182	199	202	198	192	166	118	217	115	103	191	155	300	168	111	103	198	146	229	206	379	162	170	156	149	160	269	312	198	124	95	89	101	181	161	236	37	31	62
171	4467	TAR	14	160	175	169	107	140	128	141	120	133	68	42	90	70	144	177	97	135	117	123	121	122	125	159	150	116	93	71	78	85	80	116	115	88	50	85	50	60	100	75	109	21	17	31
44	16592	Tork	199	714	740	812	494	367	393	334	404	443	340	177	274	361	414	733	327	594	414	376	377	396	339	454	787	446	371	353	328	343	248	455	523	323	194	157	170	245	245	251	309	110	48	210
21	21366	CRM	239	778	821	809	391	595	542	479	486	382	274	240	333	327	492	586	408	532	552	510	329	531	385	687	656	402	299	310	316	325	323	439	403	274	490	441	450	421	781	876	1890	127	106	329
71	13744	CRM	115	308	339	338	284	312	274	322	236	273	222	126	213	193	202	258	187	373	221	218	196	234	215	268	296	263	223	230	197	207	219	340	340	230	432	481	481	370	909	755	1631	69	74	70
100	10383	CRM	221	222	238	228	235	282	255	223	322	222	198	131	206	198	191	255	163	304	198	184	177	227	172	284	269	233	208	197	172	168	171	259	283	193	397	395	353	276	234	259	360	306	64	250
123	8666	CRM	164	202	197	240	190	264	225	189	269	184	142	78	134	137	173	217	181	261	217	185	158	250	186	319	277	182	164	161	158	107	112	152	180	132	278	184	215	226	229	219	316	173	25	414
135	7702	CRM	64	207	231	197	147	149	93	139	110	146	87	74	105	105	109	137	134	150	150	140	96	136	106	155	153	133	101	104	99	107	86	181	138	113	269	193	191	150	459	416	1536	33	45	28
4	36306	Tekay	72	594	597	686	406	324	325	286	346	371	267	267	275	301	481	502	419	488	439	462	376	429	404	459	579	399	283	256	316	246	294	373	375	341	834	833	842	967	7407	4505	5940	302	302	1336
8	28673	Tekay	35	424	444	510	123	182	113	128	148	207	108	87	174	143	262	320	292	231	259	289	216	290	256	278	258	174	108	108	112	114	114	130	197	147	689	658	707	676	7519	5834	5013	176	169	251
10	27040	Tekay	49	389	446	538	261	229	244	204	272	273	221	166	218	228	333	415	285	357	303	378	287	355	317	303	412	281	205	213	224	182	209	234	284	226	633	691	679	699	5639	4067	4216	230	177	468
16	24801	Tekay	352	344	405	375	626	610	640	592	622	558	591	270	613	571	375	573	335	1066	407	435	377	382	446	576	570	825	581	502	612	595	518	776	980	634	1152	1043	1041	1034	317	272	343	158	266	441
53	15623	Tekay	30	241	237	290	59	80	71	76	75	92	84	59	97	96	139	147	151	131	130	208	145	164	172	162	211	104	63	79	61	89	65	80	99	96	405	395	423	375	3615	3296	2692	83	101	155
61	14998	Tekay	268	229	193	266	285	384	330	362	340	250	337	146	333	386	200	315	182	631	233	251	195	234	238	297	342	472	349	328	359	417	347	409	478	344	835	662	744	757	149	180	345	54	190	352
77	13094	Tekay	24	258	228	286	132	125	107	105	121	155	108	75	125	116	166	253	159	184	165	174	193	193	179	146	181	155	111	114	101	106	106	123	139	125	322	301	338	338	2644	1581	1952	111	89	380
89	11636	Tekay	80	284	282	384	306	267	256	214	274	276	169	142	211	272	311	339	270	350	211	265	224	269	254	231	347	228	203	205	218	198	178	193	272	199	250	289	267	267	446	343	489	57	175	671
35	17734	Retand	110	430	437	452	353	300	381	408	351	299	401	307	357	308	375	546	386	640	445	436	449	435	427	559	555	618	395	319	365	434	426	608	620	414	293	351	280	276	549	520	802	206	53	58
48	16261	Retand	83	398	441	397	331	332	334	294	272	273	348	245	421	263	340	540	401	596	377	397	371	457	345	534	527	591	353	269	355	386	372	544	541	370	234	323	278	211	541	506	826	194	31	19
137	7687	pararetrovirus	104	319	345	467	84	360	257	458	249	84	45	23	69	65	211	180	217	48	325	244	258	249	259	304	309	100	39	260	53	70	28	138	159	67	55	44	60	60	113	208	321	38	26	315
6	29641	EnSpm CACTA	121	1203	1281	1207	703	670	761	610	665	560	456	321	552	465	698	935	688	790	810	824	745	861	739	945	1082	857	582	476	475	480	492	780	864	527	483	519	508	476	1042	751	1098	348	25	166
97	10545	EnSpm CACTA	87	361	308	419	314	260	267	282	330	236	181	168	217	239	251	258	246	388	274	225	237	256	251	308	313	342	232	219	246	210	187	375	306	196	173	156	126	143	182	134	236	60	45	301
20	21752	MuDR Mutator	476	533	550	517	469	587	594	534	598	408	433	350	468	454	424	631	454	662	496	519	437	440	487	641	637	685	456	333	441	397	399	618	713	449	608	482	471	444	712	577	1000	75	26	67
104	9958	5S_rDNA	197	444	349	342	192	212	301	287	237	571	82	58	169	89	118	276	188	133	102	267	204	180	194	348	224	237	168	177	243	133	227	185	149	154	45	125	78	190	570	593	802	64	42	12
1	281282	Satellite	2137	14270	15736	14422	6026	8514	8529	7421	7390	4770	6002	2773	8020	4689	6191	8647	6594	10755	7833	8209	7083	8326	7385	9238	9668	8651	7607	6132	7274	6455	5640	10459	9815	7022	2235	1539	1447	1093	936	824	1562	165	1442	356
