# Supplementary figures and images for: Expansions and contractions of repetitive DNA elements reveal contrasting evolutionary responses to the polyploid genome shock hypothesis in Brachypodium model grasses
Source: Front Plant Sci. 2024 Jul 10;15:1419255. doi: 10.3389/fpls.2024.1419255 (PMC11266827; doi:10.3389/fpls.2024.1419255)

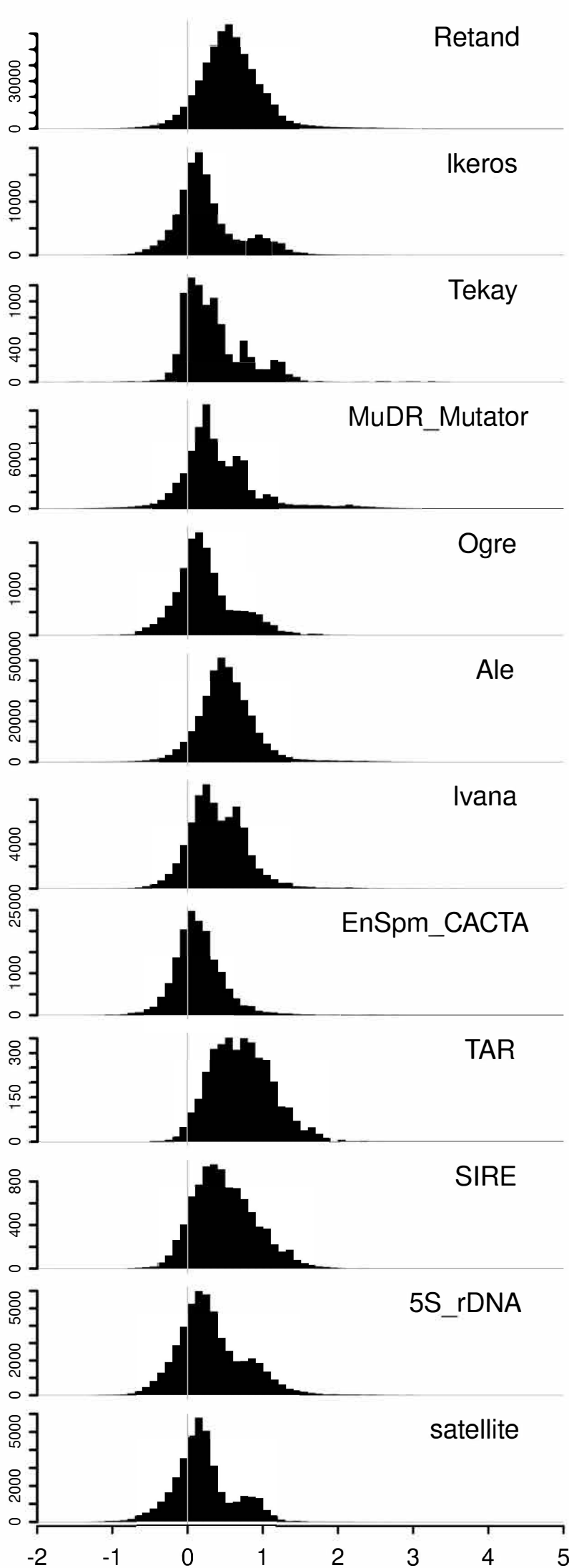

Supplement: Supplementary Figure 1 — Geographical distribution of the studied 44 Brachypodium samples. (see Table 1 , Supplementary Table S1 ). Colour codes for taxa and symbol codes for ploidy level (diploid: circle, tetraploid: triangle, hexaploid: square) are indicated in the corresponding charts. (A) B. mexicanum. (B). B. arbuscula, B. boissieri, B. distachyon, B. hybridum, B. rupestre, B. stacei. (C). B. phoenicoides, B. pinnatum, B. retusum, B. sylvaticum. [file DataSheet_1.zip › Data Sheet 1/Supplementary Figure S3.pdf]
